# Spatial distribution characteristics and influencing factors of traditional villages based on geodetector: Jiarong Tibetan in Western Sichuan, China

**DOI:** 10.1038/s41598-024-62666-z

**Published:** 2024-05-22

**Authors:** Yunzhang Li, Wenling Fan, Xiaowen Yuan, Jingya Li

**Affiliations:** 1 College of Architecture and Environment, Sichuan University, Chengdu, 610065 China; 2 School of Fine Art, Northwest University for Nationalities, Lanzhou, 730030 China

**Keywords:** Environmental impact, Environmental impact, Sustainability, Environmental social sciences

## Abstract

Jiarong Tibetan is a regional group with distinctive regional characteristics and possess precious traditional village resources. Studying the spatial distribution and influencing factors of traditional villages is of vital significance for the protection and renewal of villages and the revitalization of cultural heritage. Nevertheless, due to the fact that the Jiarong Tibetan inhabited area has not been clearly defined for a long time, there is a lack of holistic discussion on the distribution features and driving mechanisms of traditional villages in this region. In order to fill this research gap, the paper is the first to break away from the existing county administrative divisions to define the study area. Moreover, the analysis is carried out by using the nearest neighbor index, kernel density, GoeDa and Geodetector, etc. for traditional villages at national-level and provincial-level. The results show that the spatial distribution of the traditional villages of Jiarong Tibetan is characterized by typical aggregation, with the core intensive area in Danba County and the sub-core intensive area in the central and northern parts. The results of factor detection show that the spatial distribution pattern of the traditional villages is the consequence of the synergistic effect of multiple factors, and the interaction effect is significantly enhanced. The economic level and climatic conditions play a controlling role, and population, elevation, intangible cultural heritage and rivers also have notable effects. The findings of study can offer scientific guidance and suggestions for the inheritance and development of traditional villages in Jiarong Tibetan settlement area.

## Introduction

Traditional villages are the most representative form of life in settlements^1^, commonly found in the vast rural areas of China. As a carrier of human activities and culture, it contains people's ancient wisdom of adapting to and transforming nature^2^, and carries residents' nostalgic memories. The traditional villages discussed in this paper have been officially approved by the government and have a clear definition, i.e., villages formed in an earlier period, possessing richer cultural and natural resources, and having some historical, scientific, artistic, economic, and social values. By definition, traditional villages are closely linked to cultural heritage and should be paragons of villages across the country, deserving of focused protection. As of April 2023, China's Ministry of Housing and Urban–Rural Development, the Ministry of Culture and other departments have announced 6 batches of 8,155 Chinese traditional villages. Sichuan Province has published 1,165 provincial-level traditional villages.

At present, cultural heritage protection has become a world issue. From the perspective of the change of conservation object, the expansion from single cultural relics and architectural protection to the delineation of protected areas by the surrounding environment has been accomplished^3^. After the millennium, society’s emphasis on spiritual civilization has drawn scholars' attention to intangible cultural heritage^4,5^. On the other hand, the iterative updating of digital technology has also led cultural heritage research to the track of multidisciplinary collaboration and integration^6,7^. In the meantime, with post-industrial era coming, old industrial parks are facing the problem of transformation and renovation, and industrial heritage revitalization research^8^ has gradually become a special field. It is still the cities, towns and villages^3^ that receive the most discussion. In the case of a large agricultural country like China, traditional villages are used as an entry point to explore the revitalization of rural cultural heritage. Not only is it of great significance at the strategic level of rural revitalization, but it can also benefit the grassroots, promote rural production and boost rural tourism.

The Tibetans in China mainly live on the Tibetan Plateau, with distribution in Xizang, Qinghai, Sichuan, Yunnan, Gansu and other provinces. Differences in topography and geomorphology have prompted the customary scholarly division based on region^9^, roughly including the Weizang people who live on the Qiangtang plateau and South-Tibet, the Anduo people who are nomadic on the Qinghai Plateau, and the Kangba people who settle in the high mountains and deep valleys of western Sichuan. Influenced by the established impression, the Jiarong Tibetan distributed in the Dadu and Min River basins have long been included under the concept of the Kangba, resulting in lacking a systematic compilation of their own cultural underpinnings, and even the scope of their settlement has never been clearly defined. Not to mention the study of their settlement pattern and cultural heritage value in the whole region.

With regard to research on the spatial distribution of villages, most of the current studies concentrate on the more economically developed eastern and southeastern coastal areas^10,11^. There are also some discussions on special geomorphological zones^12,13^, and southern multi-ethnic settlement areas^14,15^. However, China's Tibetan areas are located deep in the western border, sparsely populated, and inconveniently accessible. Because of the high difficulty of field investigation, it is hard for most scholars to understand Tibetan areas comprehensively and objectively, and it is almost impossible to establish a wide-area systematic study. Meanwhile, Jiarong Tibetan, as one of the ethnic branches, the related research suffers from the dilemma of polarization of subjects. One is that the Jiarong traditional villages are categorized in the Sichuan Tibetan region or Kangba region, resulting in the Jiarong region being hidden in wider macroscopic scopes. The second is that only exploring a few or a single village^16,17^ in the same county makes the study limited and not universal. All of above limitations ultimately led to the fact that few previous studies have discussed the formation mechanism and causal factors of the distribution of Jiarong Tibetan villages. Therefore, it is crucial to conduct targeted research on the spatial distribution and driving forces of traditional villages by considering the Jiarong Tibetan as individual subjects.

With the rapid growth of the Western Development Policy since the twenty-first century, the future of the traditional villages of Jiarong Tibetan and its cultural heritage has been challenged. For one thing, after the Wenchuan Earthquake in 2008, the reconstruction and relocation of Jiarong traditional villages have caused a shift in the traditional landscape and cultural atmosphere. For two, environment damage and the rise of tourism economy have led to hollowing out, aging and homogenization of villages^17^. Third, community participation, public interest organizations, and local businesses are far less vocal under the current conservation system^18^.

**Figure 1:**
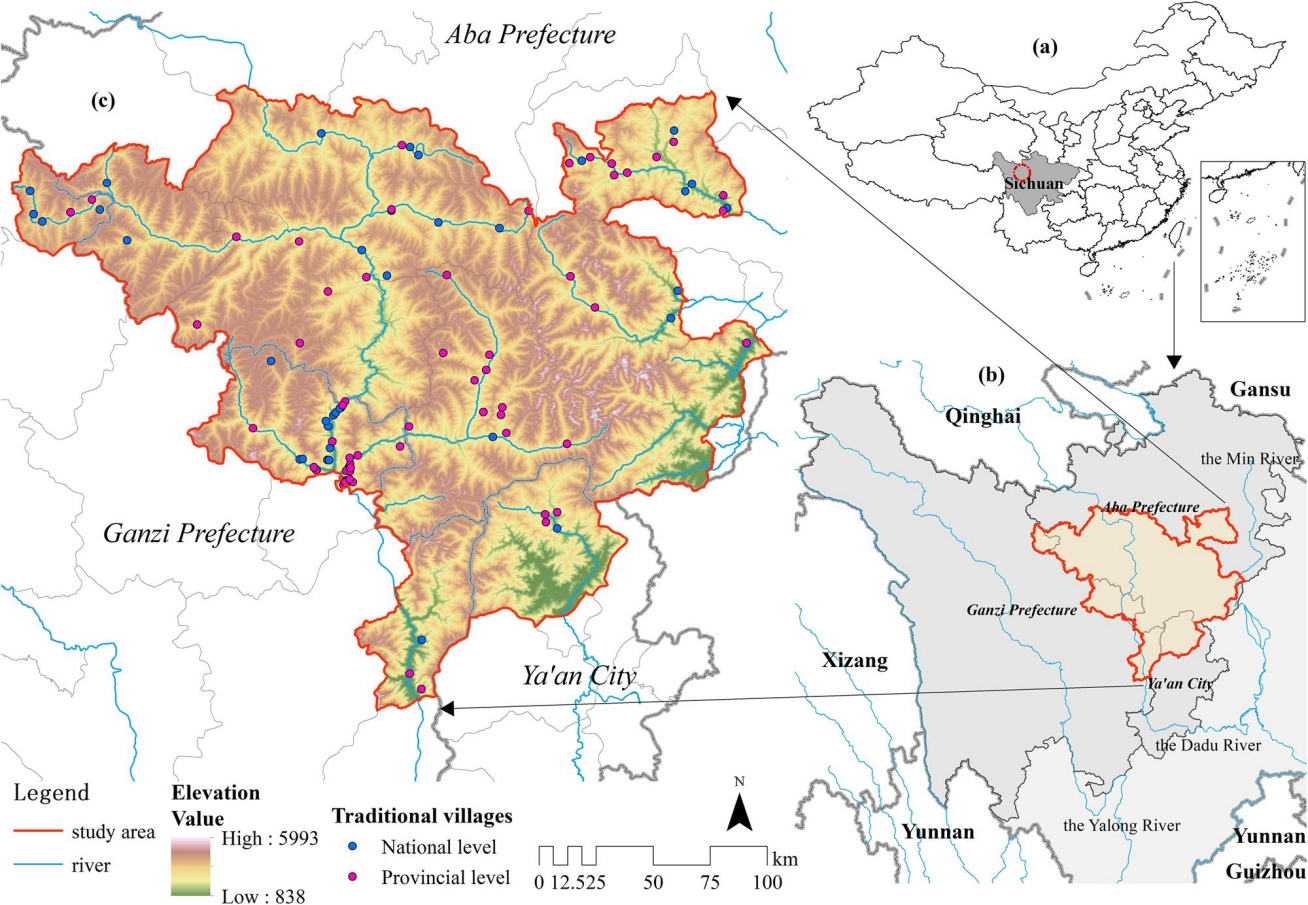
Location of the Jiarong Tibetan inhabited area. Maps were drawn by authors, using ArcGIS 10.4.1 (Environmental Systems Research Institute, USA. https://www.esri.com/).

As a result, this paper focuses on the whole area of Jiarong Tibetan inhabited area (Fig. 1) and explores the spatial distribution characteristics and influencing factors by using national and provincial traditional villages. It not only supports the preservation and renewal of the traditional villages of Jiarong Tibetan, but also complements rural cultural heritage research in ethnic areas and provide references for similar plateau areas. This study is dedicated to solving 3 problems. (1) Identifying the settlement areas of Jiarong Tibetan beyond the administrative limitations of the county. (2) Using ArcGIS, analyze the spatial distribution features of the traditional villages. (3) Using Geodetector, investigate key factors affecting the spatial differentiation of the traditional villages and summarize the mechanism of action.

## Literature review

### Research on cultural heritage and rural cultural heritage

Cultural heritage is the product of human beings' productive life in the past. Because of its epochal nature, once the original environment is destabilized or destroyed, cultural heritage can no longer be sustained^19^. Depending on the form of existence, it can be divided into tangible cultural heritage, which exists in physical form, and intangible cultural heritage, which is transmitted through oral or movement. As such, traditional villages with both physical artifacts and human practices are one of the manifestations of cultural heritage.

Western scholars were pioneers in the field of cultural heritage research, dating back to the Enlightenment^3^. At this time, scholars reported high passion for archaeological artifacts and buildings from the ancient Greek and Roman periods and devoted themselves to restoration work^20^. Until the nineteenth century, cultural heritage continued to be primarily studied in terms of historic buildings. Theoretical research has been characterized by the emergence of “stylistic restoration”^21^ represented by Eugene Viollet-Le-Duc and “anti-restoration”^20,21^ represented by John Ruskin. There was a heated debate between the two sides on whether or not architectural restoration should be fully restored to the form of the previous era. But eventually, Camillo Boito and others advocated the concept of “restoration based on history”^22^, which was widely recognized by the academic community. The core concept is that architectural heritage has its own historical and artistic continuity, so the current situation must be respected. Restoration should not artificially alter or add to the appearance, and the evidence and process of restoration needs to be documented on an article-by-article basis^21,22^.

The system of cultural heritage research was gradually improved in the twentieth century, and developed towards the trend of standardization of statutes and broadening of objects, with rural cultural heritage becoming popular in the later period. Early on, the two industrial revolutions vigorously promoted urbanization of Western countries, leading to the erosion of urban cultural heritage surfacing first^3^. Countries around the world have deliberated and introduced documents^21^, such as the *“Athens Charter”* (1933), the *“Venice Charter”* (1964), and the *“Charter of Machu Picchu”* (1977). In particular, the idea that cultural heritage protection should take into account the traditional environment^23^ was first put forward by the “Venice Charter”, and that historic neighborhoods and towns should be regarded as protected objects. With the promulgation of the *“Convention concerning the conservation of the world cultural and natural heritage”* in 1972^24^, the connotation of cultural heritage was clearly defined, i.e., historical artifacts, historical buildings (groups) and sites of human culture. Then historical cities, towns and villages with traditional architecture and integrated into nature are also logically included in the study.

Stakeholder transformation in rural cultural heritage conservation practices is also an important finding. Traditionally, cultural heritage management should be dominated by officials^18^, such as city, county, and village governments and expert groups. Nevertheless, Diana et al.^25^ and Ekici et al.^26^ found that appropriately allowing local small businesses, public interest organizations, and residents to join can prevent government-business alliances, reduce funding risks. De Luca et al.^27^ stated that the creation of bottom-up organizational structure will be the innovation management model of the future. This topic has triggered subsequent in-depth discussions on community participation^28^, sense of place^29^ and more.

Entering this century, the spiritual core of cultural heritage has become further explored, and the issues of traceability, revitalization and transmission of intangible cultural heritage^4,30^ have garnered much attention. Extracting historical stories, myths and heroic deeds known to the public from them can not only stimulate people’s sense of identity^30^, as well as promote national unity and social stability. Besides, it is conducive to the integration of cultural heritage into modern life. Common approaches such as establishing heritage parks^31^, developing rural heritage tourism^32^ and creating ethnic villages^33,34^. Additionally, technological advances have led to emerging research directions. With the help of artificial intelligence^35^, virtual reality^36^, augmented reality^37^, machine learning^38^ and other methods, traditional heritage is endowed with virtualization and socialization features, thus enabling digital heritage^7,36^, model prediction^35,38^, and intangible cultural heritage interactions, which is a new way of exploring the access to informatisation of rural cultural heritage.

In summary, the research process of cultural heritage is evolving with the times. Compared to this, China's Tibetan regions have not discussed in sufficient depth in this regard, and those focusing on the Jiarong Tibetan are even rarer. Current research still mostly stay in architectural heritage^39^ and localized tourism^32,33^, and there is a paucity of meso- and macro-explorations. Therefore, analyzing the overall distribution pattern of the traditional villages of Jiarong Tibetan will open up perspectives for the conservation of cultural heritage in high-altitude areas.

### Research on the spatial distribution and driving forces of villages

Since the twentieth century, traditional village research has made a series of theoretical achievements. After the publication of the first list in 2012, it has steadily become research hotspot. Nowadays, the spatial distribution studies have become an important research branch. Most of the existing studies have a macro perspective, unfolding in cities or provinces. There are remarkable literature outputs in Hubei, Guangdong^40–42^, Jiangsu and Zhejiang^10,11^, which are rich in traditional villages, and some discussion is found in Jin et al.^43,44^ in the Central Plains and Yun et al.^14,15,45^ in the Southwest. In recent years, some scholars have embarked on cross-regional studies, targeting a particular landform type or watershed. Li et al.^12^ and Liu et al.^13^ studied the Karst region and the Tibetan Plateau, respectively, while Feng et al.^46^ and Xu et al.^47^ explored the Yellow River coast, and Dong et al.^48^ analyzed the nine watersheds.

With regard to influencing factors, the most common are topography, hydrology, soil type, vegetation, transportation, GDP, population and ethnicity^49,50^. Some studies supplemented social factors, such as the buffer circle of central city^45^, number of intangible cultural heritage items^10,51^, the output value share of three industries^43^. For the research methods, mathematical and statistical methods are introduced, mainly Moran's I, LISA, kernel density, Geary's C, standard deviation ellipse and so on^52^.

Meanwhile, with digital technologies such as ArcGIS and CIM becoming more and more mature, the exploration about the driving mechanism of spatial distribution is getting deeper. Based on ArcGIS, Geodetector, Multi-scale Geography Weighted Regression (MGWR) to carry out the study of spatial differentiation^15,53,54^, geographic pattern^44,51^, and spatio-temporal evolution^46,55,56^ of traditional villages, which facilitates the identification with the core influences. These are currently effective means for quantitatively analyzing the distributional characteristics and formation of traditional villages.

In contrast, the study of traditional villages of Jiarong Tibetan is lagging behind. First, the research scale is narrow. Studies are mostly still centered on the dwellings, historical buildings, and public spaces in traditional villages^57,58^. A few involve meso-perspective, generally choosing 3–4 villages and exploring morphological features^59^, landscape diversity^16^, and cultural heritage protection^17,60^ and so on. Next, holistic research is lacking. The study area is mainly a localized area, and heavily concentrated in famous tourist counties such as Danba County and Markang City^16,17,59^, with very few multi-county studies. Thirdly, there has been no in-depth exploration of the mechanisms that have shaped their settlement patterns. That is also a pitfall of the unresolved question about the settlement range of Jiarong Tibetan. To summarize, the whole-area study on traditional villages of Jiarong Tibetan is still blank, and relevant research is implied in a few southwest studies. Therefore, it is necessary to make a systematic study on the distribution characteristics of traditional villages and its driving mechanisms by taking Jiarong Tibetan settlement area as a single region, which will be an essential advance in the study of settlements in ethnic areas.

### Study area and data source

#### Determination of the Jiarong Tibetan inhabited area

Currently, scholarly representations of the Jiarong Tibetan inhabited area mostly list counties. However, historical activities of the ethnic groups did not follow the present-day administrative regions^61^, and it would be inaccurate to limit Jiarong Tibetan to the districts formed in modern era. In the 1980s, Fei Xiaotong pointed out that “historically formed ethnic areas” should be the target^61,62^, i.e., areas with similar ethnicity, culture, and social types due to being in the same geographical environment. Hence, it is a primary challenge for this paper to judge the settlement areas of Jiarong Tibetan based on their history and origins.

“Jiarong” was originally a place name, and was first recorded in Chinese vocabulary by Zhuang Xueben in the Republic of China^63^. But according to Tibetan historical records, when the Tubo garrison the Dadu River and Min River in the Tang Dynasty, it already called the surrounding area of Mount Morduo (Danba County) for “Jiarmu Chawa rong”^64^. Where “Jiarmu” means the Mount Morduo, “Chawa” means the area, “rong” means suitable for farming and pastoralism. Since then, “Jiarong” has been gradually used to refer to the people living here. So, the term itself has the meaning of settlement area. From the perspective of historical development, the Jiarong Tibetan formed in the process of ethnic integration over thousands of years. Before the Tang-Bo War (623–850 A.D.), the upper reaches of the Dadu and Min rivers were ruled by the ancient Qiang, and many tribes were entrenched there. After Tubo's eastward campaign, Han and Tubo cultures spread here one after another^65,66^. The aborigines here have gone through many rounds of cultural invasions, but have not been completely assimilated by any one ethnic group. Instead, they were catalyzed by a number of factors and grew into a regional group with a relatively independent language, customs, and beliefs, which is now known as the Jiarong Tibetan.

At the same time, the reputation of the 18 Jiarong Tusi has risen, and a number of studies have confused it with the concept of Jiarong Tibetan^67^. The 18 Jiarong Tusi refers to the 18 local chiefs with military and political power, hereditary hierarchies and the policy of unification of politics and religion, that were formed successively from the Yuan Dynasty to the middle of the Qing Dynasty (1271–1840 A.D.) in the Jiarong settlement area. In reality, the jurisdiction of the Tusi extended far beyond the settlement area, as the emperor would often grant additional land to loyal chiefs. And after historical investigation, it is found that some of the Tusi's families were not Jiarong Tibetan at all^64^, such as *ShenBian*'s (Luding County) genealogy recorded as Mongolian, and *MingZheng* (Kangding City) is Kangba Tibetan. Therefore, the Jiarong Tusi is one of the considerations, but it still needs to be filtered with discretion.

**Figure 2:**
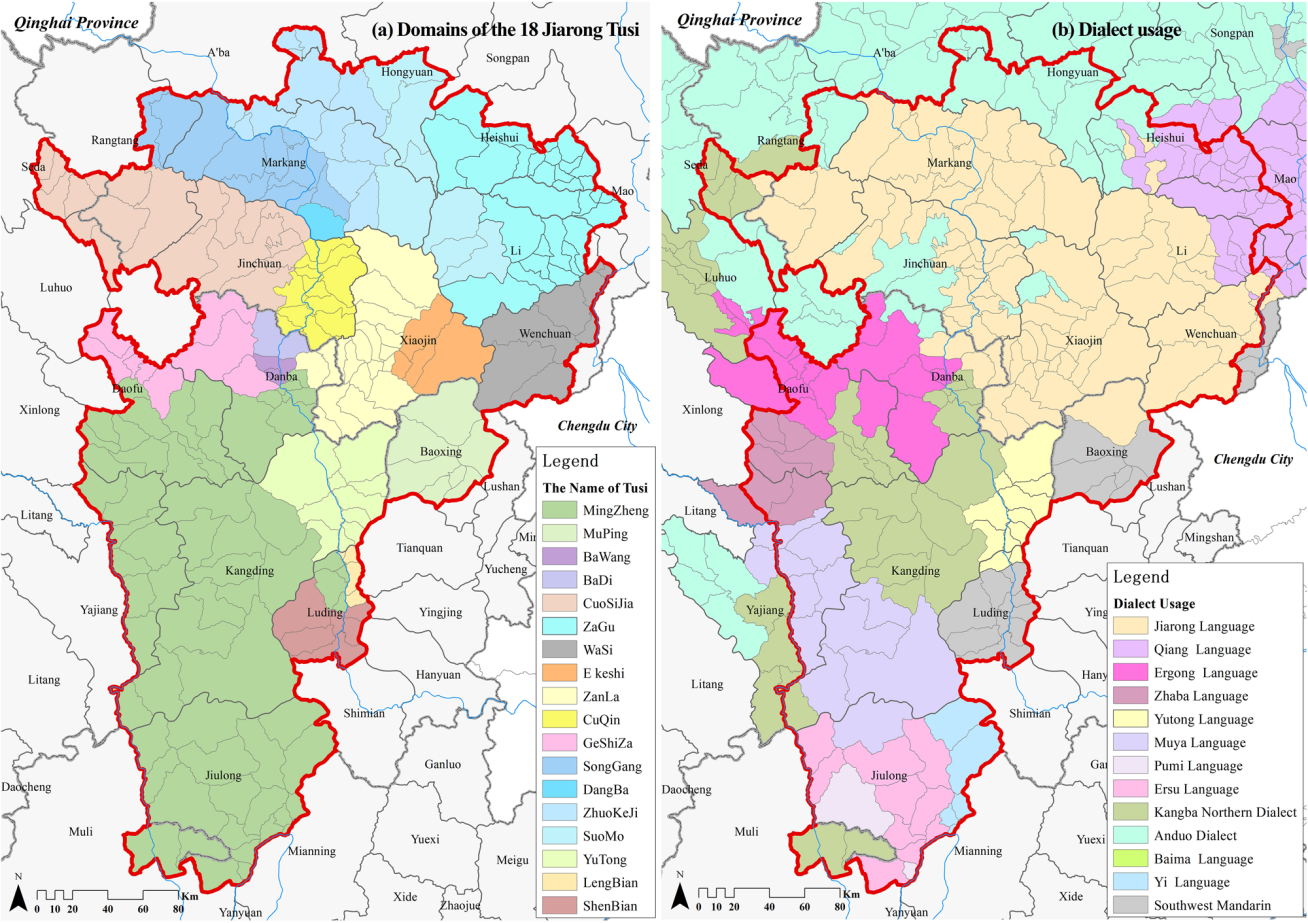
Domains of the 18 Jiarong Tusi and dialect usage in neighboring areas. Maps were drawn by authors, using ArcGIS 10.4.1 (Environmental Systems Research Institute, USA. https://www.esri.com/).

To summarize, it should be more appropriate to start from the historical area, language and culture to conduct the judgment of the Jiarong Tibetan inhabited area. This paper draws Jiarong Tusi's dominions (Fig. 2a) and dialect usage (Fig. 2b) after referring to the historical materials such as *the History and Records of the Jiarong Tibetan*, *Language Atlas of China*, *the Records of the Qiang in Aba Prefecture*, and the records of various Prefectures and Counties^64,65,68^, etc. Then, combined with the cultural identities of residents obtained from the interviews. Through superimposed analysis, the study area is finally formed, with boundaries accurate to the village.

**Figure 3:**
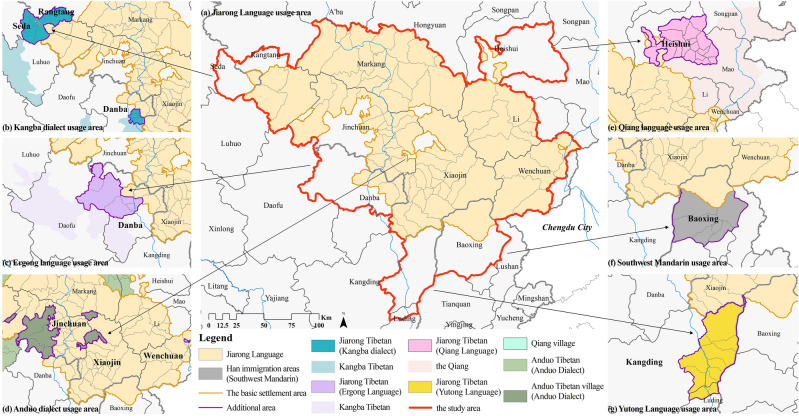
Determination process of Jiarong Tibetan settlement area. Maps were drawn by authors, using ArcGIS 10.4.1 (Environmental Systems Research Institute, USA. https://www.esri.com/).

There are four specific principles of determination. (1) Considering the regions where the Jiarong language is spoken as the basic settlement area and expanding on it (Fig. 3a). (2) Emphasizing the identification of inhabitants. It mainly involves regions that do not speak Jiarong language but still consider themselves to be Jiarong Tibetan (Fig. 3b, c, e, g), such as Heishui County (Qiang language), Seda and Rangtang counties (Kangba language), Danba County (Kangba language, Ergong language), Kangding City and Luding County (Yutong language). (3) Emphasizing the scope of historical settlement. It mainly involves regions where large numbers of Han and Hui moved in due to modern policies, such as Baoxing County (Fig. 3f). (4) Recognizing that ethnic activities are somewhat inclusive. It mainly involves the scattered villages inhabited by the Anduo Tibetan and Qiang (Fig. 3d).

#### Overview of the study area

The study area occupies the edge of the Western Sichuan Plateau in the southeast of the Tibetan Plateau, which is the transitional zone to the Sichuan Basin. The main landform is mountainous, with a wide range of altitudes, and the terrain is northwest high and southeast low. There are as many as 60 peaks over 5000 m, cutting the river valley into pieces. The meso-subtropical monsoon climate is the base zone^69^, showing a distinctly mountain climate. The valleys are mild all year, with heavy rainfall in summer, and the semi-alpine areas are slightly cooler, with clear division between wet and dry. Also, some pastoral areas have alpine climate, with no summer and long winter, but plenty of sunshine.

The Jiarong Tibetan inhabited area is simultaneously at the fringe of the Tibetan, Qiang, and Han, and is part of the Tibet-Yi corridor^61^. With the characteristics of multi-ethnic integration, multi-cultural convergence and multi-faith collision, it has nurtured traditional villages with precious culture, architectural art and folkloric skills. It is an essential part of the Chinese cultural heritage.

It covers 12 counties in Ganzi Prefecture, Aba Prefecture and Ya'an City in Sichuan Province, containing 105 towns and 730 administrative villages, with a total area of about 39,300 km^2^. There are 43 national traditional villages and 57 provincial traditional villages, totaling 100. Table 1 shows that there are also other nationalities distributed, but the Jiarong Tibetan dominate, with only 4% of the traditional villages being the Anduo Tibetan and 1% being the Han. Considering the complex causes of traditional villages, we will select the influencing factors from both natural and social environments (Table 2) on the basis of previous research and the reality of the study area.

**Table 1:** Ethnic distribution of traditional villages in Jiarong Tibetan settlement area.

Type	Ethnic group	Number	Proportion (%)	Traditional village name
1	Jiarong Tibetan	95	95	Except for the following two types
2	Anduo Tibetan	4	4	Akeli village (Jinchuan County), Mucheng village, Daping village, Molong village (Xiaojin County)
3	Han	1	1	Liangtai village (Xiaojin County)

**Table 2:** Detailed sources of data.

Type	Data source	Precision
Traditional villages data	1. Ministry of Housing and Urban–Rural Development of the People’s Repulic of China (https://www.mohurd.gov.cn/)	–
	2. The People’s Government of Sichuan Province (https://www.sc.gov.cn/)	
Physical geographic data	Resource and Environment Science and Data Center (https://www.resdc.cn/)	500 m
Climate data	1. The National Tibetan Plateau Center (https://data.tpdc.ac.cn/home)	1 km
	2. The National Earth System Science Data Center (Loess Plateau SubCenter) (http://loess.geodata.cn/index.html)	1 km
Vegetation data	MODIS datasets-MOD13A3 (https://search.earthdata.nasa.gov/search)	1 km
Socio-economic data	1. The 7th Population Census Bulletin	Town
	2. The 2022 Yearbooks of counties, government websites	Town
Road data	Gaode Map	–
Intangible cultural heritage data	1. The State Council of the People’s Republic of China (https://www.gov.cn/)	County
	2. The People’s Government of Sichuan Province (https://www.sc.gov.cn/)	

#### Data source

The data used in this paper were obtained from authoritative websites, see Table 2 for details. Firstly, the list of traditional villages was published by the official website, and then its latitude and longitude were acquired from Baidu Map, which was converted by ArcGIS to form a dataset of traditional villages. Second, topographical and river data were obtained from 500 m Digital Elevation Model (DEM), and vegetation coverage was calculated from 1 km Normalized Difference Vegetation Index (NDVI). Climate data were all derived from annual average values using multi-year data from 2000–2022. Third, population data were taken from the 7th population Census Bulletin, economic data were obtained from the 2022 yearbooks of counties and government websites, and road data were crawled by Gaode Map. The lists of intangible cultural heritage are from the websites of the China and Sichuan Province governments, respectively. In this case, the road density used fishnet tool, which requires the creation of a fishnet grid with a unit of 1 km^2^, then calculating the length of the road within each grid, and finally summing up. The per plot net income reflects the total net income that farmers receive per unit of land, which is calculated from the per capita net income of each township. Intangible cultural heritage items can be linked to counties based on declaration units. In addition, all of the traditional village assignments are realized by extract values to points tool.

## Results

### Spatial distribution characteristics of traditional villages

#### Basic forms of spatial distribution

The nearest neighbor index (R) and the coefficient of variation (CV) are used to analyze the basic layout of traditional villages of Jiarong Tibetan. Table 3 shows that the R value of both national and provincial traditional villages is < 1, so the spatial layout is clustered. Which is the smallest for all traditional villages, only 0.57. It suggests that the distribution of villages as a whole exhibits strongest cohesion, which may result in larger variations from county to county. The values of CV are similarly all > 64%, proving the clustering of villages. Among them, national-level traditional villages have the largest CV of 121.61%, indicating that they might be particularly congregated in a certain area.

**Table 3:** R-value and CV-value in the distribution of traditional villages of Jiarong Tibetan.

Grade	R-value	CV-value (%)	Distribution form
All	0.57	113.62	Cluster group
National level	0.70	121.61	Cluster group
Provincial level	0.71	94.02	Cluster group

#### Density analysis

**Figure 4:**
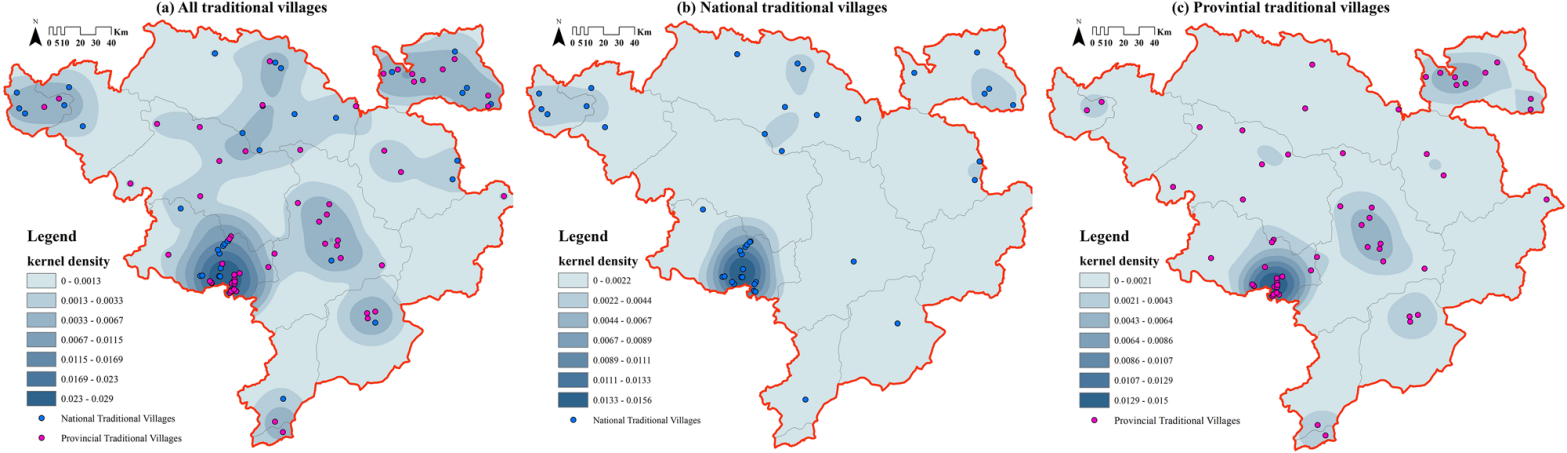
Kernel density analysis of traditional villages of Jiarong Tibetan. Maps were drawn by authors, using ArcGIS 10.4.1 (Environmental Systems Research Institute, USA. https://www.esri.com/).

The overall kernel density analysis illustrates (Fig. 4a) that one significant density core is formed in the southwest of the study area, accompanying the dispersal of sub-density cores, and no core in the east. Specifically, Danba County is the concentrated distribution area of traditional villages of Jiarong Tibetan. The next three areas are more independently distributed, located in Heishui County, Seda County and Rangtang County, Xiaojin County and Baoxing County. As for the remaining areas, they are spread out sporadically.

Consideration of traditional villages at each level reveals that national traditional villages also form a remarkable density core in Danba County (Fig. 4b), which is vastly denser than in other counties. And there are 3 sub-density cores located in Seda County, central Markang City and eastern Heishui County. The significant density core of provincial traditional villages is slightly larger (Fig. 4c), covering the extended range from Danba County to the border zone with Jinchuan and Xiaojin counties. Meanwhile, there are 2 sub-density cores, respectively in central Xiaojin County and western Heishui County. Notably, tertiary cores occur in Baoxing, Seda, Luding counties, and Yutong area, but all of them are scattered and small in extent.

#### Geographic concentration

The results in Table 4 show that the actual geographic concentration (G) of traditional villages is all greater than the geographic concentration of the average distribution (G0), indicating that the traditional villages of Jiarong Tibetan are centrally distributed. The high and low G value show that traditional villages are concentrated in different regions with various degrees of concentration. For example, the highest value is 50.24 for national-level traditional villages and the lowest value is 40.73 for provincial-level ones.

**Table 4:** Geographic concentration and imbalance index of the distribution of traditional villages of Jiarong Tibetan.

Grade	G-value (actual geographic concentration index)	G0-value (geographic concentration index under balance distribution)	Imbalance index
All	42.54	28.87	0.55
National level	50.04		0.67
Provincial level	40.73		0.56

#### Imbalance index

**Figure 5:**
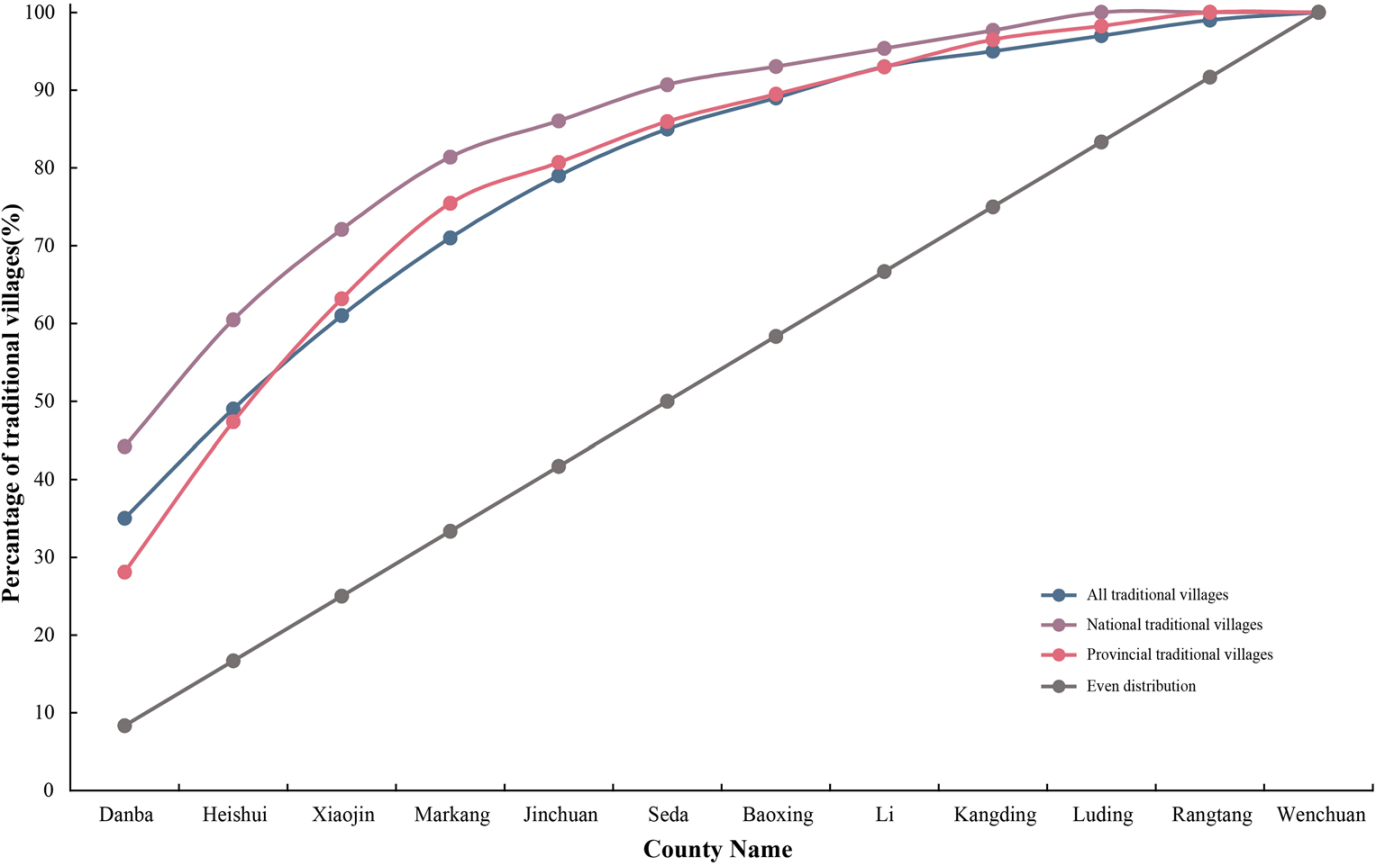
Lorenz curve of spatial distribution of Jiarong Tibetan traditional villages. The image was drawn by authors, using Microsoft Office16 Excel (https://www.office.com/).

As can be seen from Table 4, the imbalance index is all, provincial and national in descending order. It illustrates that the national-level traditional villages are the most unequally distributed, while all and provincial ones are closer to each other in some areas, but still not evenly distributed. The Lorentz plot (Fig. 5) is verifying the above analysis.

### Factors influencing the spatial distribution of traditional villages

#### Elevation and topography

**Figure 6:**
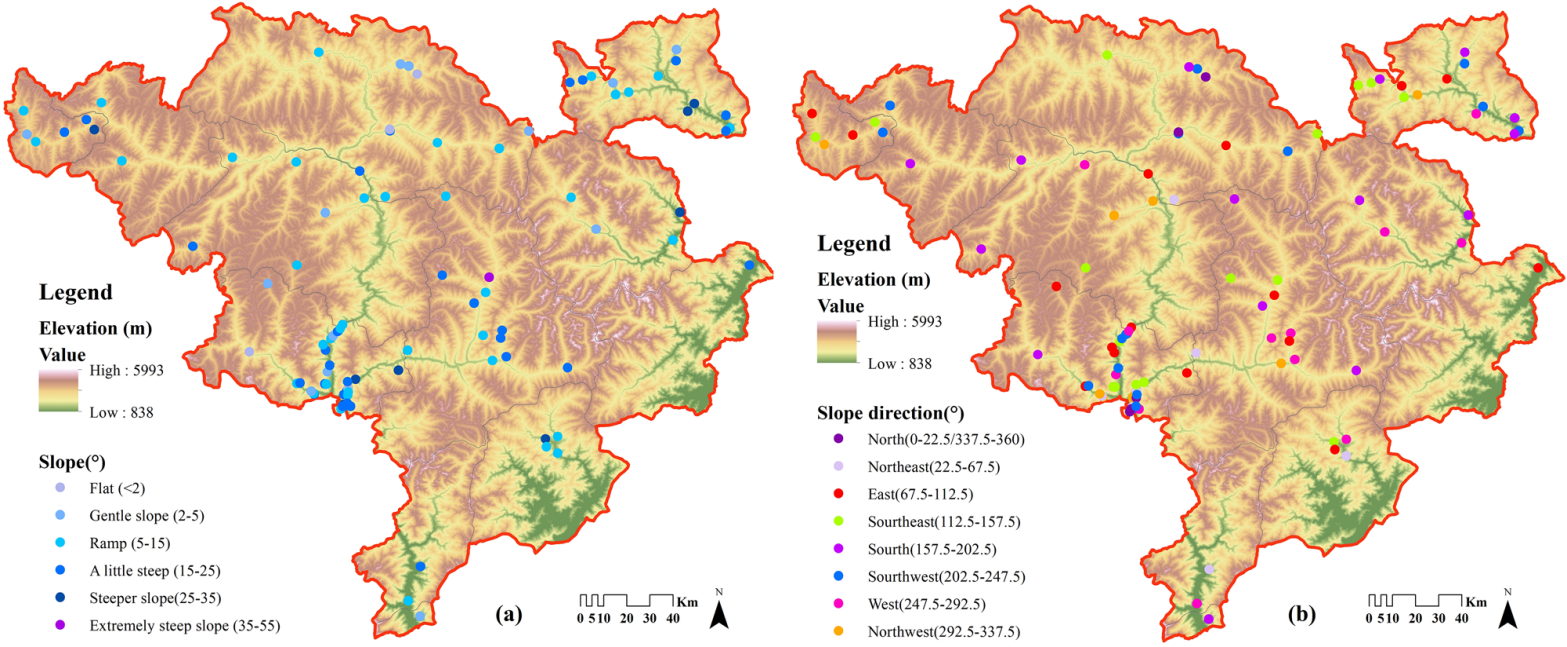
Distribution of slope and slope direction of Jiarong Tibetan traditional villages. Maps were drawn by authors, using ArcGIS 10.4.1 (Environmental Systems Research Institute, USA. https://www.esri.com/).

The traditional villages of Jiarong Tibetan are mostly distributed in river valley flat dams or mid-mountain terraces with better natural conditions, located between 1407 and 3943 m in elevation (Fig. 6), 99% of which are at high altitudes above 1500 m. Generally, hypoxia begins when people are above 2000 m, and symptoms of hypoxia are evident if the altitude increases to 3000 m. However, more than 80% of Jiarong Tibetan traditional villages are above 2000 m, of which 67% and 19% are at 2000–3000 m and 3000–4000 m respectively. This suggests that the Jiarong Tibetan prefer to live in high altitudes where oxygen is scarce.

**Figure 7:**
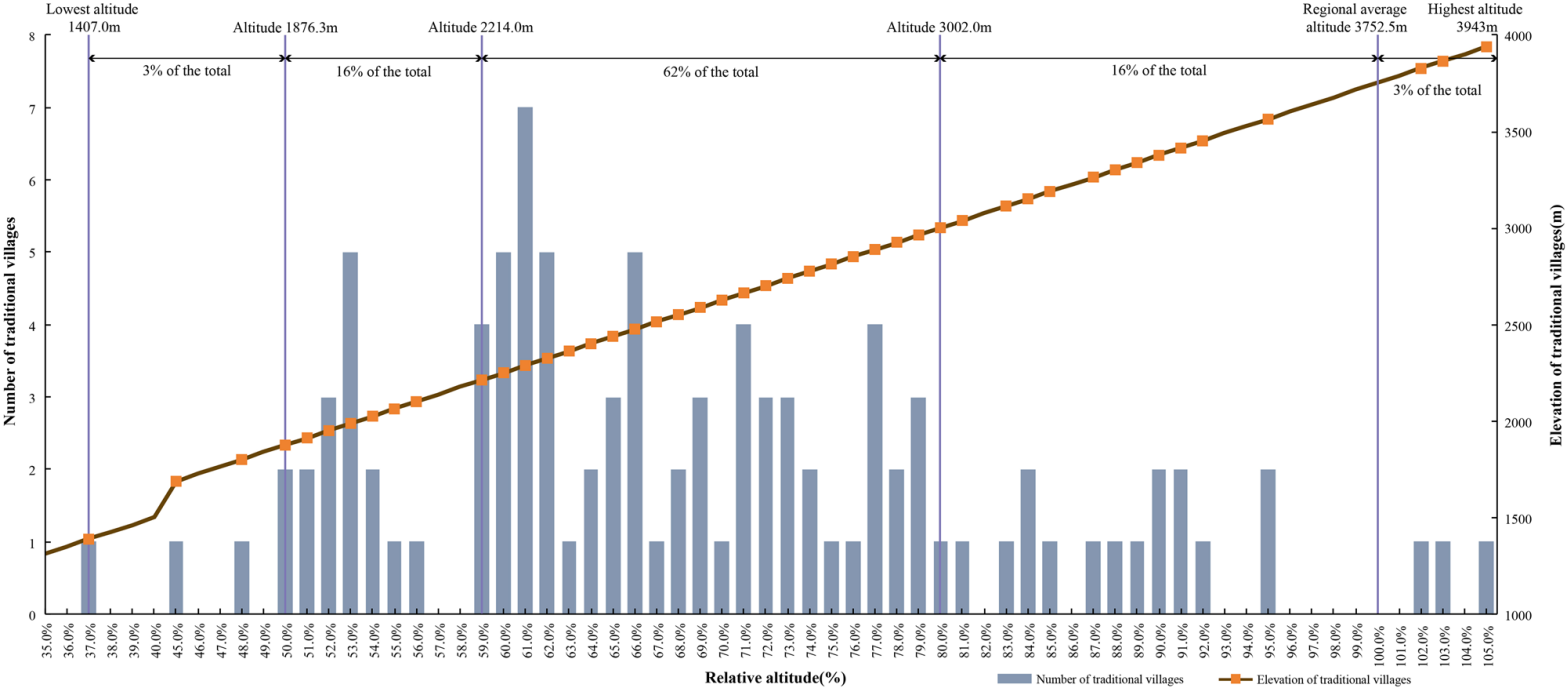
Relative altitude of traditional villages of Jiarong Tibetan. The image was drawn by authors, using Microsoft Office16 Excel (https://www.office.com/).

This paper analyses the relative position of villages in relation to the plateau on which they are located, using relative altitude as a reference. Figure 7 shows the calculation of the ratio of each village elevation to the average elevation of study area (3752.5 m). The values show that 62% of the traditional villages have relative altitudes of 59–80% (2214–3002 m), and 16% each have a relative altitude of 50–59% (1876.3–2214 m) and 80–100% (2214–3752.5 m). It further indicates that Jiarong Tibetans prefer the mid-alpine areas, and rarely choose to live in the more demanding conditions of the high and cold mountains.

The largest number of traditional villages are located on slopes from 5° to 25°, accounting for 73%, followed by 0–5° at 18%, and the fewest at greater than 25° with only 9% (Table 5). It can be seen that most of Jiarong Tibetan traditional villages are located on slopes with steeper terrain, some of which are gently sloping. But the slope direction of traditional villages is relatively free (Table 6). Due to the latitude of the study area, the slope direction of 90°–270° can be considered as yang slope and the rest as yin slope. Then a total of 68% of the villages are on the yang slopes which are more light, warmer and more suitable for cultivation. This is in line with the traditional Chinese concept that the site of settlement should be “sitting in north and facing south”.

**Table 5:** Slope data of Jiarong Tibetan traditional villages.

Slope range/°	< 2	2–5	5–15	15–25	25–35	35–55
Number/percentage	4/4	14/14	41/41	32/32	8/8	1/1

**Table 6:** Slope direction data of Jiarong Tibetan traditional villages.

Direction	North	Northeast	East	Southeast	South	Southwest	West	Northwest
Range/°	0–22.5/337.5–360	22.5–67.5	67.5–112.5	112.5–157.5	157.5–202.5	202.5–247.5	247.5–292.5	292.5–337.5
Number/percentage	6/6	7/7	16/16	17/17	18/18	15/15	12/12	9/9

#### Atmospheric conditions

The Western Sichuan Plateau is the transition zone between the Sichuan Basin and Tibetan Plateau, with a surface undulation of 300–400 m, thus giving rise to a variety of stereo climates. The more excellent water and heat conditions are important factors for the birth and development of traditional villages in Jiarong Tibetan settlement area.

**Figure 8:**
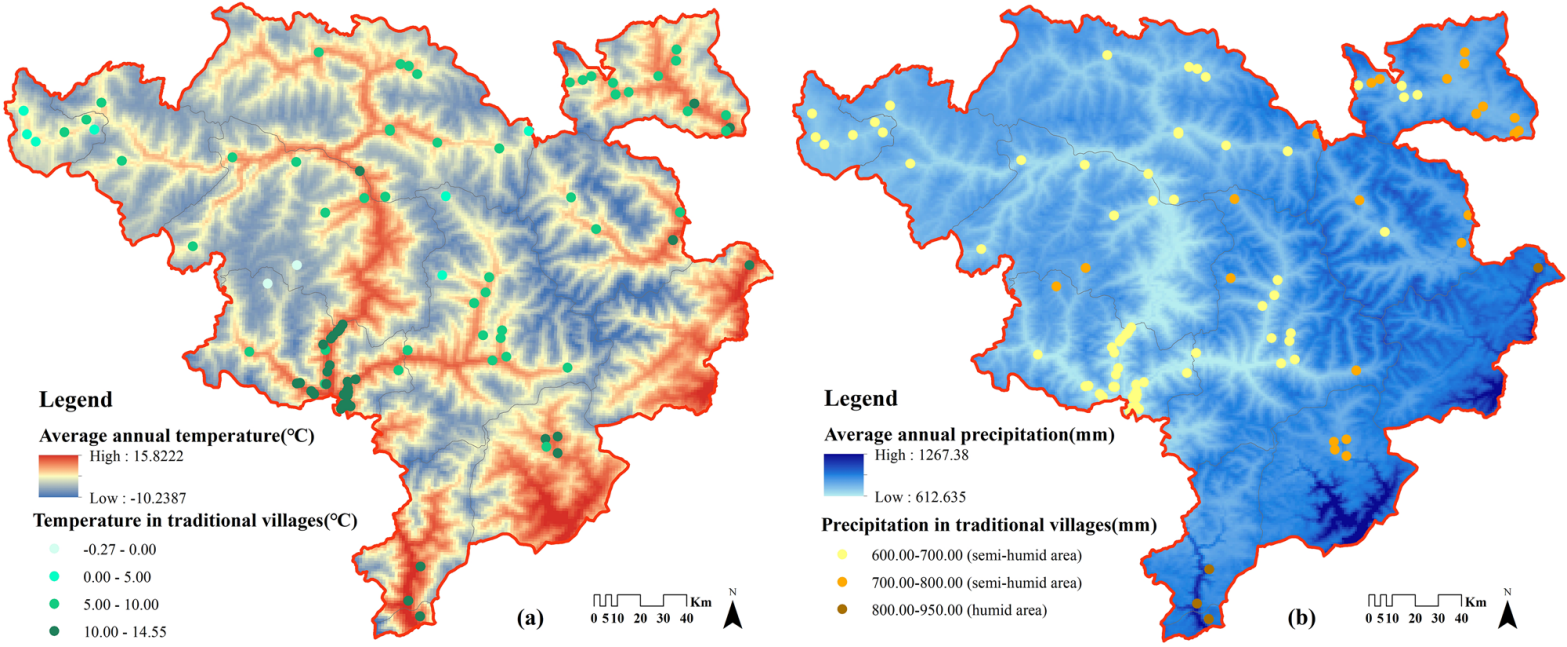
Average annual temperature and precipitation of Jiarong Tibetan traditional villages. Maps were drawn by authors, using ArcGIS 10.4.1 (Environmental Systems Research Institute, USA. https://www.esri.com/).

The vast majority of traditional villages are warmer and appropriate for semi-agricultural and semi-pastoral. There are 48% of the villages in regions where the average annual temperature is 5–10℃, and 43% is 10–15℃ (Fig. 8a). In addition, very few villages are in cold regions below 5℃, accounting for 9%. The commonality of these villages lies in the elevation of 3100 m above, and has the climatic characteristics of alpine meadows, people are mainly engaged in pastoralism, cattle and sheep industry is well developed.

In terms of average annual precipitation (Fig. 8b), there is an overall decreasing trend from southeast to northwest, with precipitation gradually decreasing as elevation increases. Traditional villages are mainly concentrated in the semi-humid area, of which 72% have an average annual precipitation of 600–700 mm. Only five villages have an average annual precipitation of more than 800 mm, all of which are located close to the Sichuan Basin in Kangding City, Luding County and Wenchuan County.

Generally speaking, solar radiation is stronger at higher altitudes, and the sunlight will have a great impact on the distribution of traditional villages. Nevertheless, it is found through statistics that the average annual sunshine hours in the Jiarong Tibetan settlement area are far less than those in Tibet, with the highest being 2275.68 h in the Xuri village, Seda County (Table 7). Only 18% of the traditional villages have more than 2100 h of average annual sunshine, mainly in the northwest. The villages located in the central part are in the range of 1800–2100 h and contain 54%, which can basically meet the needs of production and life. There are also 28% of villages less than 1800 h, belonging to less sunshine areas, with the lowest in Wenchuan and Baoxing counties, which is about 1400 h. Consequently, such climatic conditions are also consistent with the lifestyle of farming and pastoralism that has been practised by Jiarong Tibetans for thousands of years.

**Table 7:** Data on average annual sunshine hours in Jiarong Tibetan traditional villages.

Annual average sunlight hours/h	1300–1500	1500–1800	1800–2100	2100–2400
Number/percentage	4/4	24/24	54/54	18/18

#### Distance from river

The Jiarong Tibetans inhabit in the upper reaches of the Yangtze River, which has dense river networks and tributaries that run deep into various valleys. Traditional villages are mostly distributed along major tributaries of the Dadu and Min rivers. It not only ensures the convenience of water, but also undertakes the function of ancient transportation.

**Figure 9:**
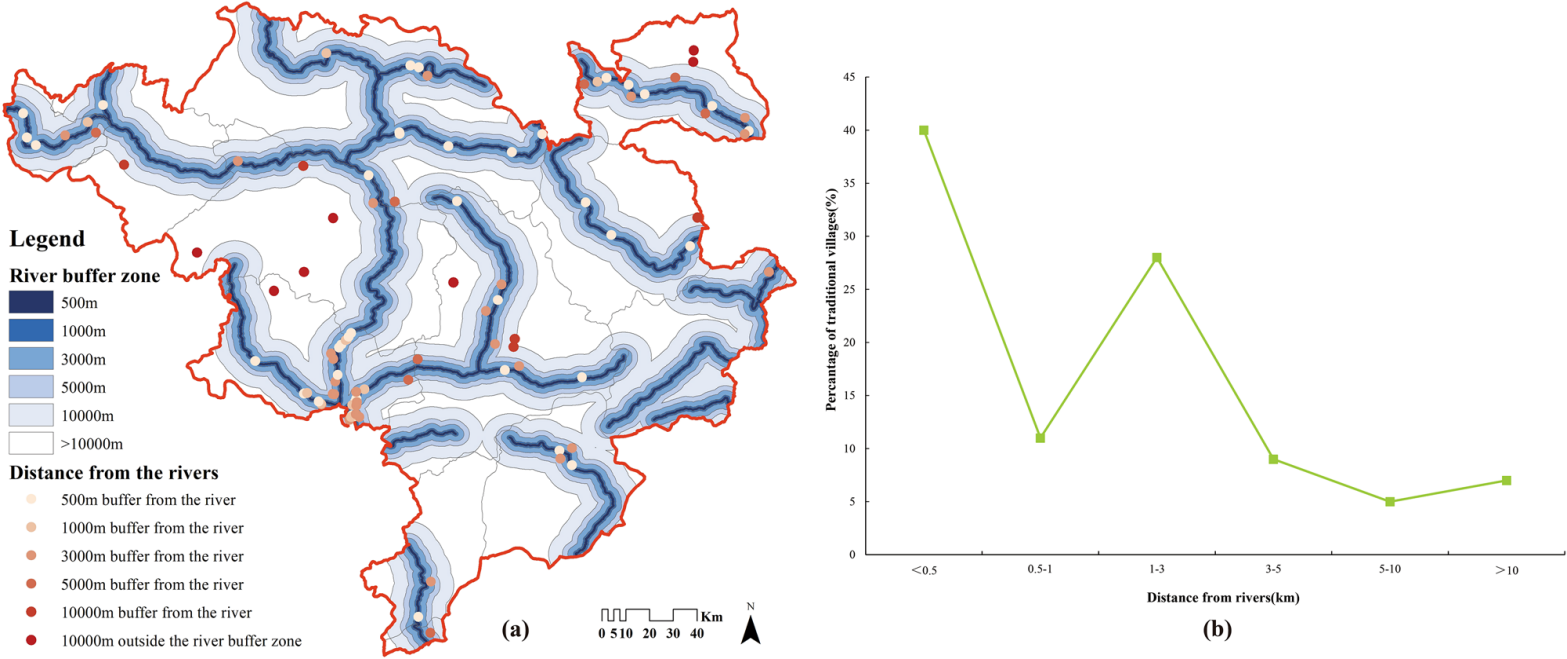
Distribution and number of river buffer zones of Jiarong Tibetan traditional villages. Figures were drawn by the authors, (a) using ArcGIS 10.4.1 (Environmental Systems Research Institute, USA. https://www.esri.com/) and (b) using Microsoft Office16 Excel (https://www.office.com/).

If the complete river network is used for analysis, the distance between all traditional villages and rivers is no more than 3.5 km, which is not very consistent with the fact. The main reason for this is that ArcGIS calculates the nearest straight-line distance, and if the river network is too dense, it inadvertently magnifies the impact of tiny streams on alpine and semi-alpine villages. Therefore, in order to more exactly reflect the water source preference, we extract the first-class tributaries as the main rivers and establish buffer zones (Fig. 9a). The results show that there are 51%, 79%, and 88% of villages within 1 km, 3 km, and 5 km radius of the river (Fig. 9b), so the vast majority of villages are located close to the rivers. In general, villages in river valleys are also equipped with the layout feature of “back towards mountain, face towards water”. While effectively blocking the cold northerly winds in winter, they also make use of the summer monsoon to regulate the microclimate for better living environment.

#### Vegetation coverage

Table 8 shows that the vegetation coverage of the region where Jiarong Tibetan traditional villages are located ranges from 0.654 to 0.995. The proportion of traditional villages with values above 0.85 is 58%, which is already more than half. At the same time, the areas with vegetation cover lower than 0.5 are mainly alpine cold desert soils and alpine meadow soils, with low and sparse vegetation, presenting plateau deserts. Therefore, the siting of traditional villages in more botanically rich locations implies that they are endowed with resource advantages. The abundant forest resources not only facilitated production, heating, grazing, etc., it was also used in the construction of dwellings, forming unique architectural structures. For example, the stone walls of folk houses in Rangtang County are embedded with wooden joist, while Heishui County adopted grain silos (Well-Frame with wood) with good sealing.

**Table 8:** Vegetation coverage in the area where Jiarong Tibetan traditional villages are located.

Vegetation coverage	0.65–0.75	0.75–0.85	0.85–1.0
Number/percentage	14/14	28/28	58/58

#### Population distribution

**Figure 10:**
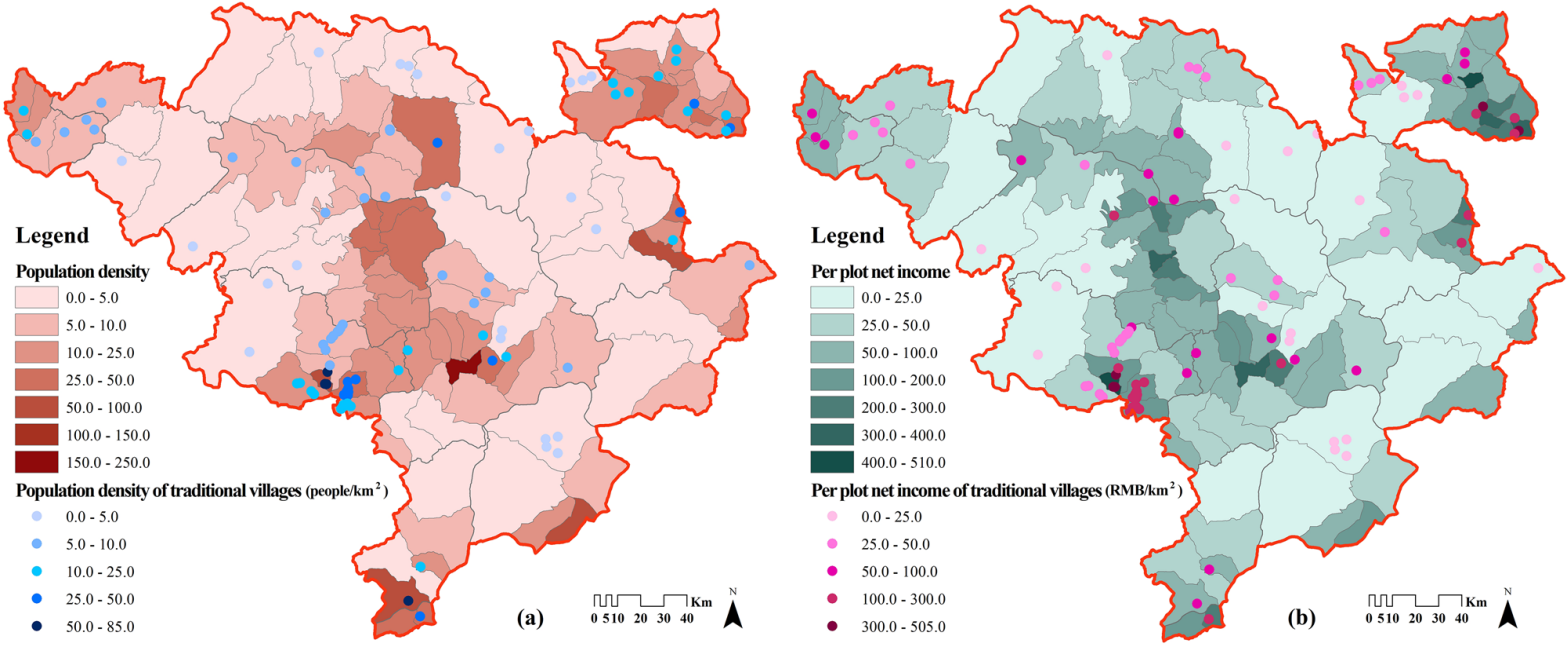
Population density and per plot net income of Jiarong Tibetan settlement area. Maps were drawn by authors, using ArcGIS 10.4.1 (Environmental Systems Research Institute, USA. https://www.esri.com/).

Figure 10a illustrates the low population density of 9.64 people/km^2^ in the Jiarong Tibetan settlement area, and a total of 82.08% of the townships are under 25 person/km^2^. Meanwhile, the density of townships where county seats are found and large tourist towns is generally high, with Luhua Town in Heishui County being smallest (13.59 person/km^2^) and Meixing Town in Xiaojin County being largest (244.82 person/km^2^).

**Figure 11:**
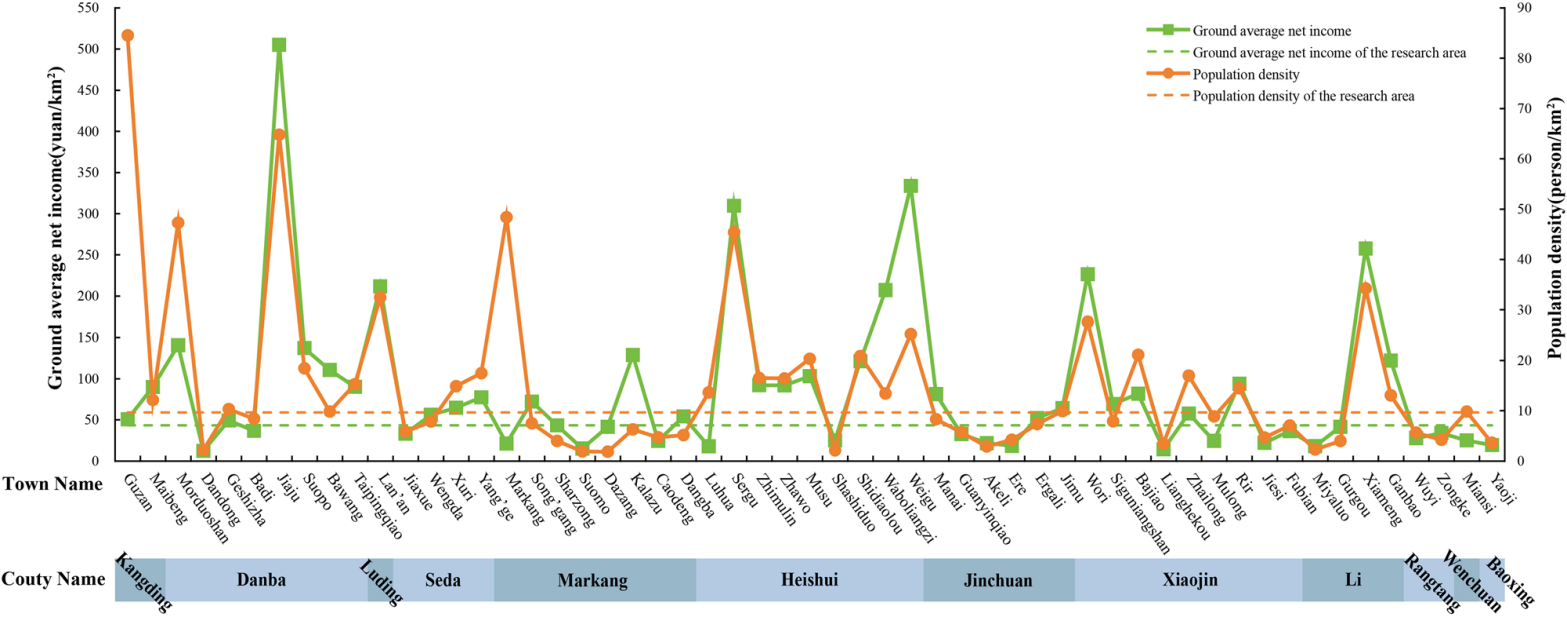
Population and income of the townships in which Jiarong Tibetan traditional villages are located. The image was drawn by authors, using Microsoft Office16 Excel (https://www.office.com/).

**Figure 12:**
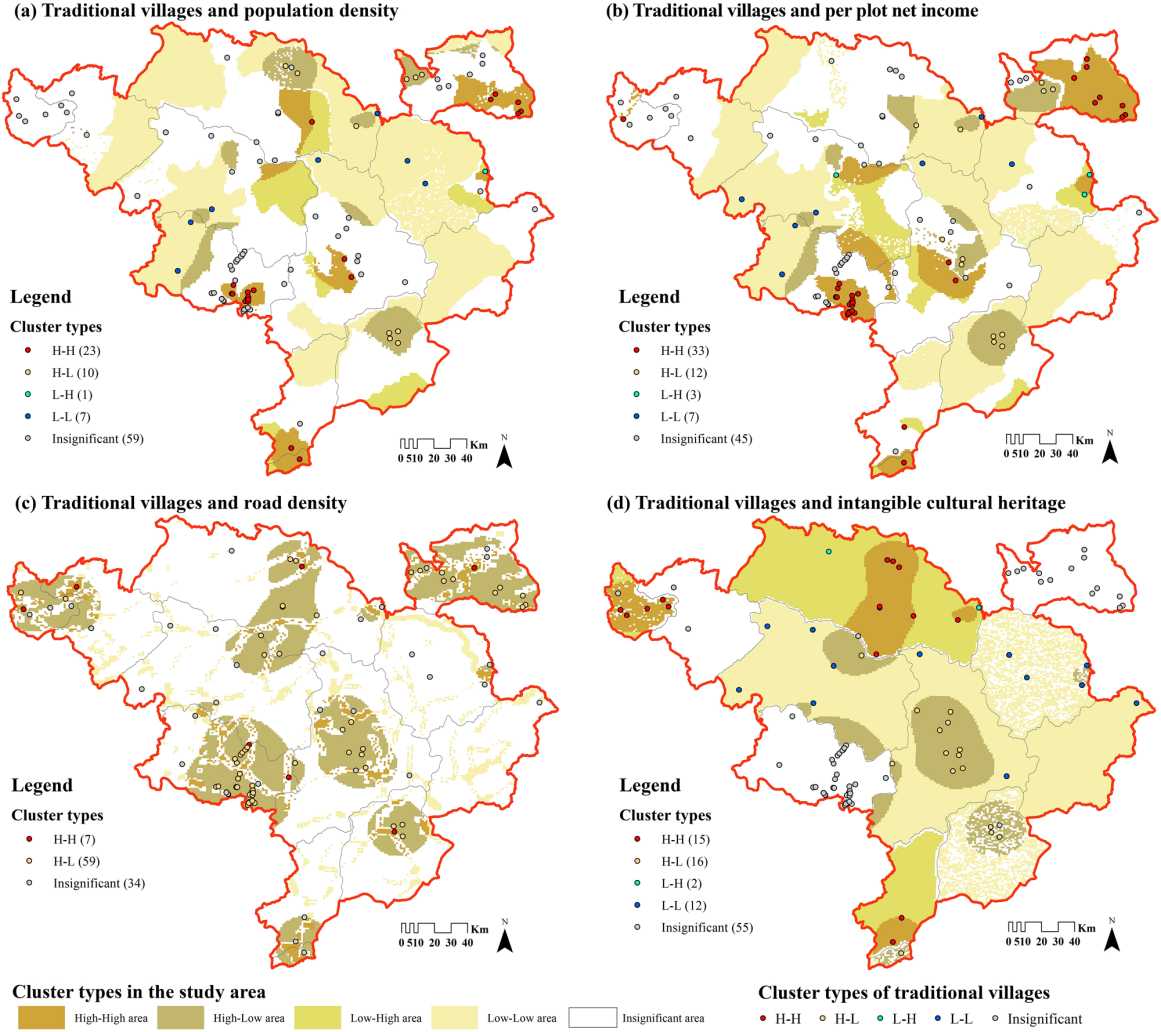
LISA cluster diagram of traditional villages and social factors in the Jiarong Tibetan settlement area. Maps were drawn by authors, using ArcGIS 10.4.1 (Environmental Systems Research Institute, USA. https://www.esri.com/) and GeoDa 1.22 (https://geodacenter.github.io/).

In contrast, the population density of the townships where traditional villages are located is at a moderately low level, with the range of 1.84–84.50 person/km^2^ (Fig. 11), and 47% of villages are less than the average (9.64 person/km^2^). These areas are farther away from the county seats, with less population flow and fewer foreign cultures entering, so there is less village destruction. On the other hand, traditional villages with higher population density are more centralized in Kangding, Luding, Danba, Markang and other counties. These areas are either in the intersection of Han and Tibet ethnic groups, or have better tourism industry and more developed economy, so the resident population is relatively larger. The results of LISA on traditional villages and population density (Fig. 12a) illustrate the positive effect of population accumulation as well, I = 0.152. There are more “High village—High population” types, which are in line with the above areas. However, the low-population areas also play a part in the preservation of traditional villages. As a result, regions with medium population density will probably be more favorable for traditional villages to survive.

#### Regional economic level

According to the calculations, the per capita net income of Jiarong Tibetans is 16,406 RMB, which is higher than the income of rural residents in Ganzi Prefecture (15,379 RMB), and slightly lower than that in Aba Prefecture (17,161 RMB) and Ya'an City (17,580 RMB) in the same year. It suggests that, compared with other areas in western Sichuan, the overall income level of Jiarong Tibetans can be categorized as medium.

Figure 10b shows that the per plot net income of the townships where traditional villages are located ranges from 12.31 to 504.99 RMB/km^2^, with notable differences from each other. However, 56% of traditional villages are higher than the average (43.31 RMB/km^2^) (Fig. 11). If the area below 75% of the average (32.48 RMB/km^2^) is regarded as underdeveloped, then 25% of villages are economically backward. It is thus found that the economies of Jiarong Tibetan traditional villages are not as bad as expected, but instead most of them have already reached above the average. The LISA clustering pattern of traditional villages and per plot net income (Fig. 12b) is also dominated by “High village—High economy”, and I = 0.329 indicates the positive effect of economy on the distribution of traditional villages. This may be due to the economic spillover effect^13^, where higher economics does not necessarily have a negative effect on its protection. However, it should not be overlooked that traditional villages in low-economic areas have formed a small amount of agglomeration, and a negative cycle of village decline due to insufficient financial support should be avoided.

#### Road transport

**Figure 13:**
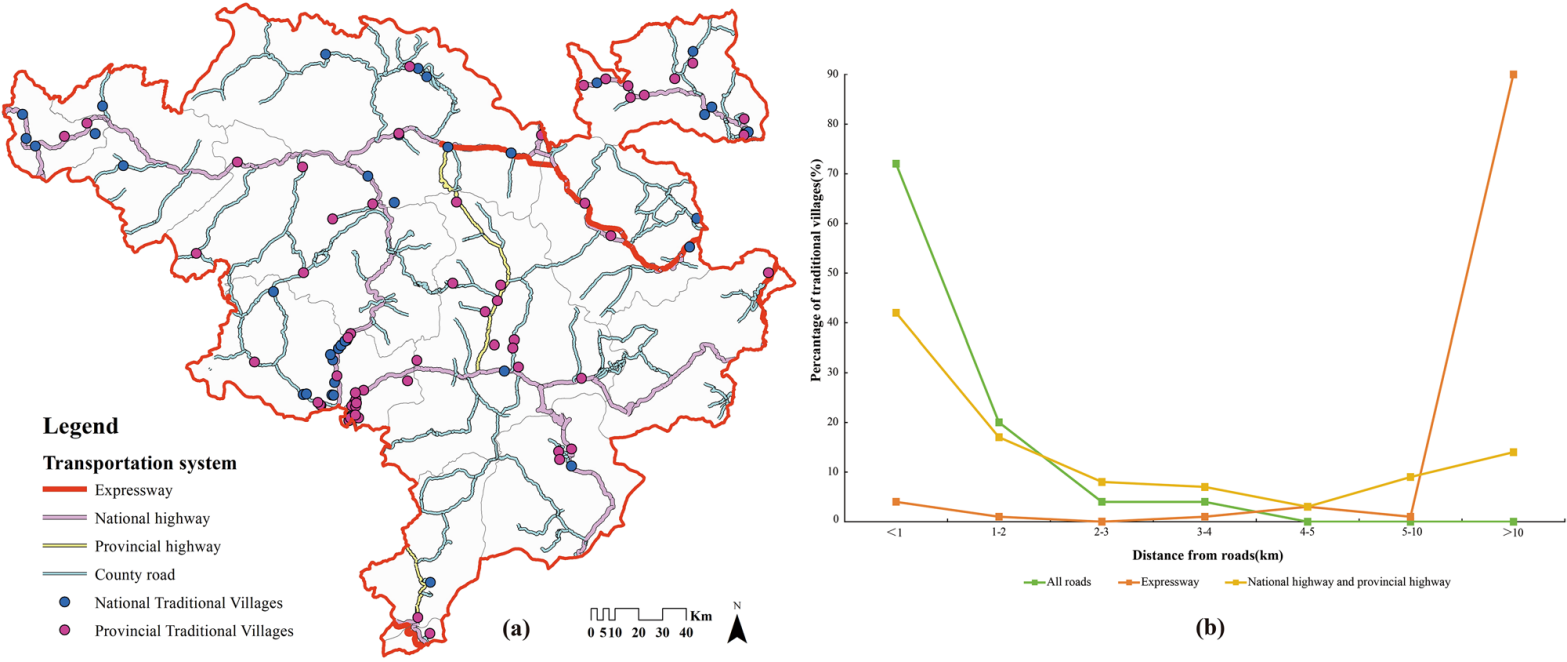
Distribution of roads and distance from traditional villages in Jiarong Tibetan settlement area. Figures were drawn by the authors, (a) using ArcGIS 10.4.1 (Environmental Systems Research Institute, USA. https://www.esri.com/) and (b) using Microsoft Office16 Excel (https://www.office.com/).

The Western Sichuan Plateau has been poorly accessible since ancient times, and the national and provincial highways were all built in the 1960s to 1970s^69^. There is no railroad in Jiarong Tibetan inhabited area so far, and the expressways can only directly reach the two Prefecture capitals (Fig. 13a). The calculated road density in the whole area is 0–8.60 km/km^2^, while that in the regions where traditional villages are located is only 0–3.61 km/km^2^, which is a big gap in both. Table 9 shows that 40% of traditional villages have a road density of 0, implying that there are no direct roads within 1km^2^ of the unit. In addition, the percentage of villages with road densities of 0–1 km/km^2^, 1–2 km/km^2^, and > 2 km/km^2^ are 24%, 34%, and 2%, proving that most villages have unsatisfactory accessibility yet. Figure 12c better visualizes this, the spatial autocorrelation pattern is dominated by “High village—Low road density”, and the I-value is − 0.103, which is a negative correlation. Therefore, traditional villages in remote areas are better preserved.

**Table 9:** Road density in the area where Jiarong Tibetan traditional villages are located.

Density of roads/km/km^2^	0	0–0.5	0.5–1	1–1.5	1.5–2	> 2
Number/percentage	40/40	10/10	14/14	29/29	5/5	2/2

Figure 13b indicates that 96% of traditional villages are within 3 km radius of all roads. However, while 90% of the villages are more than 10 km away from expressways, and 23% are more than 5 km away from national and provincial highways. Actually, county roads are an important means of connecting villages and act as capillaries. Moreover, most traditional villages are now connected by roads, but communication with outside world is still not deep. Because in reality, the vast majority of villages are reached by mountain roads, the distance traveled, the time spent, and the difficulties are far beyond theory. Furthermore, it remains to be verified whether there is close correlation with the distribution of traditional villages, taking into account that the transportation has changed dramatically in recent years.

#### Intangible cultural heritage

Traditional villages are important places for the survival, teaching and display of intangible cultural heritage. Considering that the study area is a multi-ethnic crossroads, the heritage items were screened to exclude those belonging to the Qiang, Han and Anduo Tibetans. Total 81 items (16 national, 65 provincial) were finally taken into analysis.

**Figure 14:**
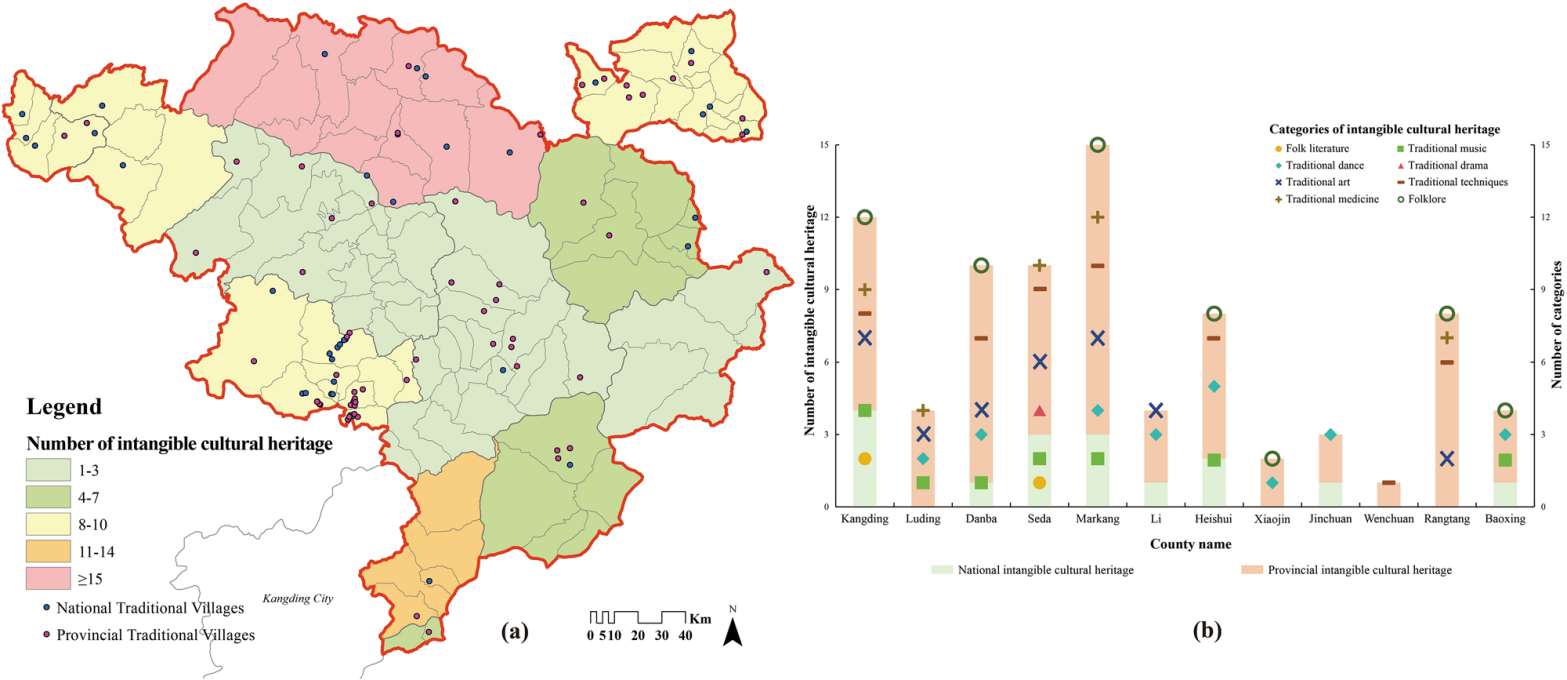
Distribution and categories of ICH in counties in which traditional villages of Jiarong Tibetan are located. Figures were drawn by the authors, (a) using ArcGIS 10.4.1 (Environmental Systems Research Institute, USA. https://www.esri.com/) and (b) using Microsoft Office16 Excel (https://www.office.com/).

Figure 14a shows that most areas with better preserved ICH are also densely populated with traditional villages, but localized areas exhibit the opposite trend. Firstly, the distribution of national-level traditional villages is positively correlated with the number of ICH, as the presence or absence of ICH is one of the evaluation indicators. That is evidenced by “High village—High ICH” in Fig. 12d. Second, Markang City is the area with the most ICH remaining at present, up to 15 items. In contrast, Jinchuan and Xiaojin counties are in a poor state, with “High village—Low ICH” and “Low villages—Low ICH” in the LISA clusters. This is likely to be a historical legacy. The QianLong Emperor of the Qing Dynasty initiated two DaXiaoJinChuan Campaigns (1747–1776 A.D.), which wiped out the CuQin TuSi and ZanLa TuSi, and dispatched a large number of garrison and immigrants to carry out drastic social reforms and reshaping of beliefs. So, it has dealt a devastating blow to the settlement pattern and folk culture of two counties, leading to the contradiction between the distribution of traditional villages and the inheritance of ICH. Thirdly, Danba, Heishui, Rangtang and Seda counties are in the third level of ICH distribution, roughly matching the density of traditional villages. Additionally, statistics difficulties make the Yutong area a special case. Currently, only accurate information on declared units is available, and it is not possible to search for towns. That is why the data of Kanding City had to be used instead of Yutong area, which may be the reason for inflating number of items.

At the same time, by analyzing the categories of ICH (Fig. 14b), it can be seen that traditional techniques, dance, art, folklore, and music are the main components of Jiarong Tibetan's ICH, and they are widely distributed. The main reason why these items can circulate steadily in villages is that they are closely related to the daily lives of Jiarong Tibetan.

### Detection of factors influencing spatial differentiation

After the above analysis, it is found that the distribution of traditional villages has spatial stratified heterogeneity, and is affected by a variety of factors, which need to be further explored. In this paper, we adopt Geodetector to first conduct single factor detection to judge whether the influence factor can explain spatial differentiation. Then, interaction factor detection is used to judge whether the influence is enhanced or weakened when the two factors work together. Each factor is numbered as elevation (X_1_), slope (X_2_), slope direction (X_3_), average annual temperature (X_4_), average annual precipitation (X_5_), average annual sunshine hours (X_6_), distance to major rivers (X_7_), vegetation coverage (X_8_), population density (X_9_), per plot net income (X_10_), road density (X_11_), distance to all roads (X_12_), and number of ICH (X13).

#### Single factor detection

The measured q-values are presented in Table 10, indicating that there are large discrepancies in the explanatory power of the influencing factors on the spatial differentiation of Jiarong Tibetan traditional villages. First of all, 4 factors failed the test of significance with *p* values greater than 0.05, suggesting that they have little or no effect and will be excluded. The reason for this is that traditional villages tend to have steeper slopes (X_2_), relatively free slope direction (X_3_), high vegetation cover (X_8_), and closer distance to all roads (X_12_), so all of these factors show high consistency, without obvious differences in distribution.

**Table 10:** Factor detection results of spatial distribution of Jiarong Tibetan traditional villages.

q statistic	X_1_	X_4_	X_5_	X_6_	X_7_	X_9_	X_10_	X_11_	X_13_	*p* value
Elevation (X_1_)	0.170396	–	–	–	–	–	–	–	–	0.005
Average annual temperature (X_4_)	↖0.520847	0.440470	–	–	–	–	–	–	–	0.000
Average Annual precipitation (X_5_)	↗0.615133	↖0.753619	0.370925	–	–	–	–	–	–	0.000
Average Annual sunshine hours (X_6_)	↖0.538798	↖0.748149	↖0.541646	0.363196	–	–	–	–	–	0.000
Distance to Major rivers (X_7_)	↗0.484937	↖0.550993	↖0.548972	↖0.550249	0.210552	–	–	–	–	0.003
Population Density (X_9_)	↖0.483860	↖0.628553	↖0.681274	↗0.712644	↗0.619352	0.321126	–	–	–	0.000
Per plot net income (X_10_)	↗0.740138	↖0.786973	↖0.826858	↖0.874272	↖0.742107	↖0.808836	0.533626	–	–	0.000
Road density (X_11_)	↗0.418132	↖0.515263	↗0.595717	↖0.528325	↖0.372969	↖0.411547	↗0.704270	0.155468	–	0.011
Number of intangible cultural heritage(X_13_)	↖0.524045	↖0.737463	↖0.641364	↖0.693171	↖0.556559	↖0.611363	↖0.687232	↖0.495777	0.352137	0.000
Slope (X_2_)	–									0.548
Slope direction (X_3_)	–									0.116
Vegetation coverage (X_8_)	–									0.315
Distance to all roads (X_12_)	–									0.086

1. Diagonal values are for single factor detection, others are for interaction detection.

2. ↖ means Two-factor enhancements, ↗ means Nonlinear enhancements.

3. When q-values are similar, nonlinear enhancement is more effective than two-factor enhancement.

Secondly, the remaining factors are ranked in descending order of explanatory power as follows: q(X_10_) > q(X_4_) > q(X_5_) > q(X_6_) > q(X_13_) > q(X_9_) > q(X_7_) > q(X_1_) > q(X_11_). The impacts of per plot net income and average annual temperature is significantly stronger than others, followed by average annual precipitation, average annual sunshine hours, number of ICH, and population density, and the role of other factors is weak. It can be seen that the regional economic level and climatic conditions are controlling factors, population affects to a certain extent. For one thing, it verifies the conjecture that the spillover of economic benefits act on the protection of traditional villages, with faster-growing regions having more funds for conservation measures to be put in place. For another, the combination of temperature, precipitation and sunshine ensure that villages are sited in places with favorable water and heat conditions. Furthermore, ICH affects the integrity of cultural environment of traditional villages, and the greater the number of items, the better it is for maintaining traditional ethos of villages. At last, the advantage of areas with medium population density is demonstrated. Because if population is too low, it leads to dispersed settlements that are difficult to centralize. Conversely, it triggers the transformation of villages into towns that will be more urbanized.

#### Interaction factor detection

The results in Table 10 indicate that the interactive explanatory power of any two factors is stronger than that of single factor, including two-factor enhancement and nonlinear enhancement. Specifically, the explanatory power of q(X_4_ ∩ X_5_), q(X_4_ ∩ X_6_), q(X_4_ ∩ X_13_), q(X_6_ ∩ X_9_), q(X_10_ ∩ X_1_), q(X_10_ ∩ X_4_), q(X_10_ ∩ X_5_), q(X_10_ ∩ X_6_), q(X_10_ ∩ X_7_), q(X_10_ ∩ X_9_), q(X_10_ ∩ X_11_) is greater than 0.7, suggesting that the per plot net income, average annual temperature, average annual sunshine hours directly affect the enhancement results. All the q-values associated with the per plot net income, except ICH, are above 0.7, which plays a prominent role. The three factors, temperature, sunshine and precipitation, which are highly correlated by themselves, also have high effects. In addition, there are slightly stronger explanatory powers for population and climate, population and river distance, ICH and climate, ICH and population economy, and elevation and precipitation.

In contrast, the influence of humanistic factors is particularly significant, with income level as the dominant driver and population and ICH as the secondary, which feeding back into the retention of the traditional villages through effective dual material and cultural support. At the same time, natural factors are the innate foundation for the birth of traditional villages, which objectively and realistically reflect the settlement tendency of Jiarong Tibetans. Climate is second only to economy. Additionally, it is worth noting that elevation and precipitation exhibit nonlinear enhancement, pointing that the superposition of geography and climate will have a larger impact on the spatial differentiation of traditional villages.

## Discussion

This paper mainly investigates the following four contents. First, the clear settlement range of Jiarong Tibetan is proposed for the first time across administrative regions, watersheds and dialect areas. Secondly, the basic distribution form of traditional villages of Jiarong Tibetan is discussed. Thirdly, the spatial distribution preferences of traditional villages on different factors are analyzed from the aspects of natural and humanistic environments. Fourth, we quantify how intensely each factor plays a role in the spatial differentiation of traditional villages, and explore the driving mechanisms.

The study focuses on a specific group at high altitude, improves the study of rural cultural heritage, and is a breakthrough in research on ethnic area settlements. It will provide theoretical and data supporting for the renewal of traditional villages and conservation of cultural heritage of Jiarong Tibetan, which is helpful to promote local rural revitalization. In the process, several findings are developed.

1. On the whole, the Jiarong Tibetan inhabited area is a channel connecting many ethnic groups, including the Kampa Tibetan, the Anduo Tibetan, the Han and the Qiang. It has prompted the evolution of Jiarong Tibetan throughout cultural integration. Similarly, the spatial distribution of traditional villages has been shaped in processes of multi-ethnic exchanges, migration and assimilation, making them precious samples like living fossils. That's exactly where the value of traditional villages lies.
2. Jiarong Tibetan traditional villages' spatial distribution is characterized by typical aggregation, which resembles to the distribution patterns of traditional villages in most parts of China^11,14^^.^ The core intensive area is Danba County, and the sub-intensive areas are mainly clustered along first-class tributaries of the Dadu and Min rivers and are relatively independent.
3. Traditional village spatial distribution of Jiarong Tibetan is not only related to single factor, but also driven by the synergy of multiple factors, and the magnitude of factor detection results can judge the main and secondary driving force. On the one hand, the Western Sichuan Plateau is characterized by numerous streams and gullies and prominent vertical zonal changes, which inevitably affect people's intention to settle down. On the other hand, the special cultural atmosphere and historical background have added more complex possibilities to the spatial distribution of traditional villages, resulting in the influence of humanistic factors exceeding that of natural factors nowadays. It differs from other studies that consider nature to play a decisive role^49,51,56^.
4. Natural factors play a crucial role in site selection, reveal the traditional residential preferences of Jiarong Tibetan, in which climate have a significant impact, and elevation and rivers have a certain effect on the distributional differentiation. First of all, Jiarong Tibetans favor areas that are warm and humid, have more precipitation, are close to water sources and are at higher altitudes. It is also largely in line with their inherent image in the literature as “tribes living in warm agricultural areas”^64,67^. Besides, because the settlement area is half-agricultural and half-pastoral, and it is in the border area where the Tibetan, Han and Qiang converge. Thus, the altitude of Jiarong Tibetan villages is not particularly high compared to other Tibetan areas (e.g., Tibetan areas in Tibet and Qinghai Province are both 4,000 m and above). The relative altitude data verifies this, with 97% of the traditional villages below the average elevation of study area. Thirdly, all natural factors are closely related and can complement each other, but not necessarily both. Take Tuyushan village in Wenchuan County as an example, it has an average annual precipitation and temperature of 850.37 mm and 12.18℃, which is very suitable for farming and living, but the only thing is that sunshine hours are only 1,330.93 h. Therefore, in order to compensate for the lack of light caused by yin slope, the village have been built on ridge terrace at 1892 m by increasing the elevation as much as possible.
5. Humanistic factors are the key to the survival and development of Jiarong Tibetan traditional villages, in which the positive effect of the regional economic level is significant, followed by population and ICH, and transportation is relatively limited. Unlike the views held by most previous studies^14,15,45,48^, this paper finds that the controversies caused by economic development need to be discussed in context, and that a certain degree of economic growth is rather beneficial to the renewal of traditional villages^13,50^.

In particular, townships with per plot net income of 43.31–200 RMB/km^2^ have the largest number of existing traditional villages, which account for 47%, while townships less than 43.31 RMB/km^2^ and more than 200 RMB/km^2^ have 42% and 11%, respectively. These are the 3 types of situations that occur in the Jiarong Tibetan settlement area, which also happens to epitomize most areas in China at the moment. Low-economy areas have preserved large numbers of traditional villages as a result of lagging development and low urbanization rates. But such a “Low village—Low economy” development model is very dangerous, since traditional villages are always at risk of disappearing due to relocation and merger. High-economy areas have seen rapid rural construction and the shrinking of traditional villages. The middle-economy areas, having certain material guarantee and not being subject to large demolition and construction, have gradually begun to demand the balance between economic growth and village preservation^44^. For example, some villages in Danba, Heishui and Xiaojin Counties are characterized by the “High village—High economy” model. The townships in which they are located have iconic tourist attractions, such as Jiaju Village and Zhonglu Village in Danba County, Dagu Glacier and Sergu Village in Heishui County, and Mount JiaJin and Mount Four Girls in Xiaojin County. Not only have clusters of traditional villages been formed, but also residents' incomes have grown considerably. The top economically ranked townships in the study area also come from this area, with highest in Jiaju Town (504.99 RMB/km^2^), second in Weigu Town (333.57 RMB/km^2^), followed by Sergu Town (309.62 RMB/km^2^) and Wori Town (226.24 RMB/km^2^).

Second, the positive role of intangible cultural heritage inheritance for traditional villages conservation has become a consensus, and there is generally a high degree of spatial consistency^15,51^. This paper finds that ICH is the cultural soul of traditional villages, which can simultaneously have strong effects with climate, economy, and population, together contributing to the development of traditional villages of Jiarong Tibetan. ICH was formed in the accumulation of long history and civilization, while people are the main body of creating culture. Attracting the population to gather and establish a certain scale of settlement is the first step. Oriented by this purpose, it corresponds to the importance of natural climatic for village siting discussed earlier. Since then, as the population continues to gather, the influence of the Jiarong Tibetan culture will also grow, gradually radiating outward to form the Jiarong cultural circle^62^. A good economic foundation, meanwhile, can change people's living needs and enhance their spiritual pursuit, thus bringing market opportunities for traditional villages and ICH preservation^26,33,51^. Examples include the establishment of ICH experience bases, transforming ancient houses into ICH exhibition halls and village museums, all of which can preserve cultural heritage in traditional villages. This improves public participation^19,27^, realizes the revitalization of traditional villages and ICH, as well as drives the rural tourism economy.

The effects of population and transportation are similar to other studies by Chen^42^, Li^12^, Xie^54^ and others. However, it should be noticed that the poor accessibility of traditional villages also maps the psychology of the ancient Jiarong Tibetans in pursuit of residential security. In ancient China, Jiarong Tusi set political power and divine power in one, were local emperors at the grass-roots level. There were frequent wars and rebellions among them in order to compete for resources. So, society from top to bottom formed the ultimate pursuit of defense performance, embodied in the village, official fortress, towers, dwellings and other aspects. Then the inconvenient transportation has become advantage, and villages situated in the high and semi-high mountains basically have a natural barrier that is easy to defend and difficult to be attacked.

## Conclusion

With reference to literature review and research interviews, this paper breaks through the existing administrative divisions for the first time and innovatively determines the scope of Jiarong Tibetan settlement area. On this basis, we analyze the spatial distribution characteristics of traditional villages from cultural geography point of view, taking national and provincial traditional villages as objects. Then, it discusses the influencing factors and driving forces of spatial distribution of villages. Research conclusions are set out below:

1. The traditional villages of Jiarong Tibetan are clearly concentrated in the southwestern of the settlement area, followed by the middle and northern parts. Danba County has the greatest number, following Heishui County, Xiaojin County, and Markang City. The calculation results of all indices suggest that traditional villages are unevenly distributed and present regional clusters.
2. There are 12 selected influencing factors, including elevation, slope, slope direction, temperature, precipitation, sunlight, rivers, vegetation, population, income, road density, number of ICH, and distance from roads. Regarding physical geography, the distribution preferences of traditional villages in Jiarong Tibetan settlement areas can be summarized as high altitudes (> 2000 m), steeper yang slopes, warm and humid, better lighting, and near-water areas with rich vegetation. On the socio-economic front, it is mainly located in districts with medium populations, moderately developed economies, rich intangible cultural heritage and poor transportation accessibility.
3. In general, it is clear that the economic level and climatic conditions play a controlling role in traditional villages distribution of Jiarong Tibetan, with population, elevation and rivers having obvious effect. Consequently, the spatial distribution of traditional villages is the result of the combined effect of humanistic factors and natural factors, and there is an enhancement effect between multiple factors, with a complex mechanism. Of which, nature determines the birth of villages, the economy feeds on the inheritance of villages, and these two coupled with other factors synergistically promote the establishment of distribution pattern. The clarification of the specific mechanism offers ways for the protection of traditional villages.

Considering that this article still has shortcomings, subsequent study could be improved. Firstly, for the sake of the holistic research, we argue that the area where the Yutong language is spoken should be the fringe of Jiarong Tibetan. But the area is academically controversial and needs further detailed justification. Secondly, during the field research, the research team found a number of equally representative ordinary villages that could be added to the study.

## Methods

### Nearest neighbor index

Generally, the spatial distribution pattern of point elements can be categorized into 3 types: random, uniform, and clustered. The nearest neighbor index (R) is a common measure^10,11^, which is the ratio of the actual closest neighbor distance to theoretical closest neighbor distance. If R < 1, it shows traditional villages are clustered distribution. If R = 1, it is random and if R > 1, it is uniform. The formula is as follows:

1 $$ R = \frac{{\overline{{r_{1} }} }}{{\overline{{r_{E} }} }},\;\overline{{r_{E} }} = \frac{1}{{2\sqrt {n/A} }} $$

where $R$ is the nearest neighbor index; $\overline{{r_{1} }}$ is the actual closest neighbor distance; $\overline{{r_{E} }}$ is the theoretical closest neighbor distance; $n$ is the number of traditional villages; $A$ is the area of the study area.

### Coefficient of variation

In statistical methods, the coefficient of variation (CV) serves to quantify the dispersion of data. When applied to geography, Tyson Polygons need to be created for each target village point first, requiring that the distance from any point inside to the target point is shorter than that to other points. The degree of villages' agglomeration is then assessed by calculating the ratio of the standard deviation of Tyson Polygons' area to the mean^43,46^. If CV ≤ 33%, it indicates uniform distribution of traditional villages, if 33% < CV < 64%, it is random, and if CV ≥ 64%, it is clustered. The formula is as follows:

2 $$ CV = \frac{R}{S} \times 100\% ,\;S = \sqrt {\mathop \sum \limits_{i = 1}^{n} \left( {S_{i} - S} \right)^{2} /n} $$

where $CV$ is the coefficient of variation; $R$ is the standard deviation; $S$ is the mean; $S_{i}$ is the area of the *i*st Tyson Polygon; $n$ is the number of Tyson Polygons, which equals those of traditional villages.

### Kernel density

Kernel density calculates the denseness of point elements and their surrounding neighborhoods^11,12^. Taking the traditional village to be valued as the center of the circle, if the closer to its center, the higher the value, then it is more densely distributed. Conversely, the sparser the distribution. The formula is as follows:

3 $$ f\left( x \right) = \frac{1}{nh}\mathop \sum \limits_{i = 1}^{n} k\left( {\frac{{x - x_{i} }}{h}} \right) $$

where $f\left( x \right)$ is the kernel density; $k\left( {\frac{{x - x_{i} }}{h}} \right)$ is the kernel function; $x$ is the location of traditional villages to be valued; $x_{i}$ is the location of villages falling within a radius of $h$ centered on $x$; $h$ is the search bandwidth ($h$ > 0); $n$ is the number of traditional villages.

### Geographic concentration index

Geographic concentration index evaluates the degree of aggregation of point elements^41,44^, which further characterizes the distribution of villages within a given region. It is necessary to assume a value of G0 when traditional villages are evenly distributed, and then calculate the actual geographic concentration (G) of traditional villages at each level. The value domain of G is [0,100]. If G > G0, it indicates traditional villages are centrally distributed, if G = G0, it is uniform, and if G < G0, it is dispersed. The formula is as follows:

4 $$ G = 100 \times \sqrt {\mathop \sum \limits_{i = 1}^{k} \left( {\frac{{x_{i} }}{T}} \right)^{2} } $$

where $G$ is the geographic concentration; $x_{i}$ is the number of traditional villages in the $i$ th county; $T$ is the number of traditional villages; $k$ is the number of counties.

### Imbalance index

Imbalance index (S) reflects how balanced the distribution of point elements is^43,56^. The value domain of S is [0,1]. If S = 0, it indicates traditional villages are evenly distributed, and if S = 1, they are all distributed within the same county. So, with higher values, the distribution is more centralized. The formula is as follows:

5 $$ S = \frac{{\mathop \sum \nolimits_{i = 1}^{n} Y_{i} - 50\left( {n + 1} \right)}}{{100n - 50\left( {n + 1} \right)}} $$

where $S$ is the imbalance index; $n$ is the number of counties; $Y_{i}$ is the ratio of the number of traditional villages in each county to the totals, in terms of the cumulative percentage of the *i*th position after sorting from largest to smallest.

### Vegetation coverage

Fractional Vegetation Cover (FVC) is a parameter that describes the condition of vegetation on the ground surface and a basic indicator of ecological environment. The value domain of FVC is [0,1], and when the value is larger, the vegetation coverage is higher. The quantile method is used for the assignment, and 5% and 95% confidence intervals are extracted^70^, which attenuates some of the errors generated by NDVI during the acquisition and transmission process. The formula is as follows:

6 $$ FVC = \frac{{\left( {NDVI - NDVI_{s} } \right)}}{{\left( {NDVI_{v} - NDVI_{s} } \right)}} $$

where $FVC$ is the vegetation coverage; $NDVI$ is the NDVI of computed image; $NDVI_{v}$ is the NDVI of purely vegetated image; $NDVI_{s}$ is the NDVI of completely unvegetated image.

### Bivariate local Moran’s I

Spatial autocorrelation means that anything is spatially correlated, and the closer the distance, the stronger the correlation^50^. Generally, it can be divided into global spatial autocorrelation and local spatial autocorrelation. Local spatial autocorrelation is more suitable for identifying the spatial aggregation and distribution characteristics of two types of elements^71^. In this paper, we will use GeoDa software to further portray the correlation between social factors and the distribution of traditional villages with the Local Indicators of Spatial Association (LISA). The value domain of $I$ is [-1,1], and as the value gets closer to 1, it indicates the stronger positive correlation. Conversely, the stronger the negative correlation. The formula is as follows:

7 $$ I_{i} = Z_{i} \mathop \sum \limits_{j = 1}^{n} w_{ij} z_{j} $$

where $n$ is the number of traditional villages; $z_{i}$ and $z_{j}$ are the variance-standardized values of observations in regions $i$ and $j$; $I_{i}$ is the local correlation between the independent variable in region $i$ and the dependent variable in region $j$. Based on the calculation results, the spatial distribution relationship between traditional villages and influencing factors can be categorized into five types, including High–High area (H–H), Low–Low area (L–L), High–Low area (H–L) and Low–High area (L–H) and insignificant area.

### Geodetector

Geodetector is mainly used to detect spatial stratified heterogeneity, which is a statistical method to reveal factors driving the dissimilarity phenomena^52^, and provide single factor detection, interaction detection, risk detection and ecological detection. The rationale is based on the assumption that if the independent variable (X) has meaningful effect on the dependent variable (Y), then the spatial distribution of X and Y should also have similarity. The effect of the two factors acting together on Y can also be detected. It requires Y to be a numerical or type quantity, but X must be a type quantity. If X is a numerical quantity, discretization is required. We adopt the natural breakpoint method and empirical judgment for hierarchical categorization of influencing factors. Therefore, when the hierarchical distribution of influencing factors (X) overlaps with the spatial distribution of traditional villages (Y), the larger the q-value is, the higher the explanatory power of X on Y, and the greater the impact. The value domain of q is [0,1]. In this paper, single factor detection and interaction detection are employed for quantitative analysis. The formula is as follows:

8 $$ q = 1 - \frac{{\mathop \sum \nolimits_{h = 1}^{L} N_{h} \sigma_{h}^{2} }}{{N\sigma^{2} }} $$

where $q$ is the explanatory power; $h = 1, \ldots ,L$ is the classification of a particular X or Y; $N$ is the number of cells in the study area; $\sigma^{2}$ is the variance of Y in the study area; $N_{h}$ is the number of cells in the class $h$; $\sigma_{h}^{2}$ is the variance of Y in the class $h$.

## Data Availability

Data are available in a public, open access repository and can be freely downloaded from the official websites, as detailed in the “Data source” section. The datasets used and/or analyzed in the current study are not publicly available because this experiment was done collaboratively and the results belong to the team, but are available on demand from the corresponding author.

## Abbreviation

ICH: Intangible cultural heritage
